# Nanosecond Laser Pulses Facilitating Efficient and Specific Cell Killing with Doxorubicin‐Loaded Gold Nanoparticles Targeted to the Folate Receptor

**DOI:** 10.1002/smsc.202400234

**Published:** 2024-12-19

**Authors:** Ilia Goemaere, Anna Cielo, Raffaella Daniele, Francesca Mastrotto, Stefaan C. De Smedt, Winnok H. De Vos, Stefano Salmaso, Kevin Braeckmans

**Affiliations:** ^1^ Laboratory of General Biochemistry and Physical Pharmacy Faculty of Pharmaceutical Sciences Ghent University Ottergemsesteenweg 460 9000 Ghent Belgium; ^2^ Laboratory of Cell Biology and HistologyDepartment of Veterinary Sciences University of Antwerp Universiteitsplein 1 2610 Wilrijk Belgium; ^3^ Department of Pharmaceutical and Pharmacological Sciences University of Padova Via Francesco Marzolo 5 35131 Padova Italy

**Keywords:** cancers, chemotherapies, drug deliveries, gold nanoparticles, laser irradiations

## Abstract

Nanoparticle‐based drug carrier systems with active targeting and a controlled release capacity are of considerable interest to bypass side effects of conventional chemotherapy. One appealing approach involves chemotherapeutic‐loaded photothermal nanoparticles, where laser irradiation can release the loaded anticancer drug while also inducing local cytotoxicity through photothermal effects. This study investigates the potential of using nanosecond‐pulsed laser light for efficient and specific killing of folate receptor (FR)‐overexpressing cancer cells in combination with FR‐targeted doxorubicin‐loaded gold nanoparticles (AuNPs). Nanosecond pulsed laser irradiation allows the induction of mechanical forces alongside thermal effects. The effect of nanoparticle concentrations and laser fluences on cytotoxicity is systematically tested, achieving near‐complete tumor cell killing under the most stringent conditions. FR targeting is confirmed using FR‐positive and ‐negative cell lines, showing that folic acid functionalization of AuNPs results in more favorable nanoparticle–cell interactions and more efficient photothermal effects. Additionally, doxorubicin could be efficiently released from the AuNPs and endosomal compartments upon laser irradiation, adding to the observed cytotoxicity. Cell killing was precisely confined to irradiated cells, leaving surrounding cells unharmed. Overall, the significant reduction of tumor cell viability following the proposed combination demonstrates this approach to be a promising step toward safer, more effective anticancer therapies.

## Graphical abstract

By combining chemotoxicity and potent photothermal effects, nanosecond‐pulsed laser irradiation of folate receptor targeted and doxorubicin‐loaded gold nanoparticles enables precisely confined cytotoxicity to the irradiated regions, leaving surrounding cells unharmed. As such, it is a step toward safer and more effective cancer therapies.

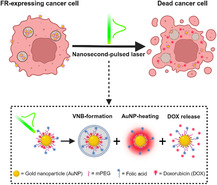

## 1 Introduction

According to the latest report from the International Agency for Research on Cancer (IARC), there were 19.3 million new cases of cancer globally and nearly 10 million cancer‐related deaths in 2020,^[^ ^1^ ^]^ making cancer one of the leading causes of mortality worldwide.^[^ ^2^ ^]^ With the aging and growing world population, these numbers are expected to significantly increase in the coming years.^[^ ^3^ ^]^ While surgical intervention, endocrine therapy, chemotherapy, and radiotherapy are currently the most efficacious commonly used treatments, there remains a need for new and improved treatments due to the incomplete removal of tumor cells, unwanted side effects, and tumors that remain refractory to treatment because of their heterogeneous nature and (acquired) drug resistance.^[^ ^4^, ^5^, ^6^, ^7^ ^]^ Some of the most promising newer treatments include immunotherapy,^[^ ^8^, ^9^ ^]^ gene therapy,^[^ ^10^ ^]^ phototherapy,^[^ ^11^, ^12^ ^]^ and combination treatments.^[^ ^13^ ^]^ Incorporating the use of nanoparticles is especially appealing, either for their intrinsic anticancer properties or as drug carriers.^[^ ^14^, ^15^, ^16^ ^]^ Nanoparticles can accumulate in tumors (i.e., passive targeting)^[^ ^17^ ^]^ and improve drug solubility, stability, circulation time, and cellular uptake, making them a versatile platform for cancer treatment.^[^ ^18^, ^19^, ^20^ ^]^ By functionalizing the nanoparticles with targeting ligands (i.e., active targeting), off‐target effects can further be reduced.^[^ ^21^ ^]^ Targeting the folate receptor (FR) is particularly attractive as it is often overexpressed by tumor cells.^[^ ^22^ ^]^

Successful loading of nanoparticles with a wide variety of chemotherapeutics has been achieved for various types of nanoparticles, ranging from inorganic to biomimetic and hybrid nanocarriers.^[^ ^23^, ^24^, ^25^, ^26^ ^]^ However, these systems have often faced relatively poor clinical efficacy,^[^ ^27^ ^]^ partly due to the inadequate intracellular release of the chemotherapeutics.^[^ ^28^, ^29^ ^]^ Therefore, controlled drug release by specific triggers, such as changes in pH, temperature, light, ultrasound, redox/enzymatic reactions, aims to overcome this obstacle.^[^ ^18^, ^30^, ^31^, ^32^ ^]^

Using light as a trigger is particularly interesting because it provides precise spatiotemporal control over the release of chemotherapeutics from photothermal nanocarriers. Gold nanoparticles (AuNPs) are well suited for this approach since they can be easily functionalized and have excellent photothermal properties.^[^ ^33^, ^34^ ^]^ AuNPs have already shown great potential for both phototherapy and the delivery of chemotherapeutics, including doxorubicin (DOX).^[^ ^35^, ^36^, ^37^ ^]^ In fact, previous studies have shown that continuous‐wave (CW) laser irradiation of AuNPs can enhance the release of the loaded agents within cells.^[^ ^12^, ^38^, ^39^, ^40^, ^41^, ^42^ ^]^ In addition, by providing extended CW irradiation, reactive oxygen species and heat, which diffuse into the tumor environment, will be produced by the AuNPs and add to the killing effect.^[^ ^43^, ^44^ ^]^ However, heat diffusion does not stop at the tumor boundaries, increasing the risk of damage to healthy tissue. Instead, using nanosecond laser pulses of sufficiently high intensity, nanoparticles can be heated instantaneously to such an extent that the liquid in its immediate environment evaporates, leading to the formation of vapor nanobubbles (VNBs). The expansion and collapse of VNBs result in distinct strong mechanical forces which can damage cells in the immediate vicinity.^[^ ^45^, ^46^ ^]^ Interestingly, as VNB formation happens at the timescale of tens of nanoseconds, heat has not the time to diffuse out of the nanoparticles, making it an essentially purely mechanical photothermal effect.^[^ ^47^, ^48^ ^]^ While a highly localized photothermal cell killing mechanism, the combination of nanosecond pulsed laser irradiation and DOX‐loaded AuNPs has not been investigated so far.

In this study, we examined the killing effect of DOX‐loaded AuNPs upon activation by nanosecond pulsed laser light. We assessed how cytotoxicity is influenced by nanoparticle concentration and laser pulse fluence. Using FR‐overexpressing SKOV‐3 cells^[^ ^49^ ^]^ and FR‐negative A549 cells,^[^ ^50^ ^]^ we also investigated the FR‐targeting capacity of AuNPs that were additionally functionalized with folic acid (FOL). Finally, we investigated the mechanism underlying cytotoxicity by analyzing nanoparticle–cell interactions, light‐triggered release of DOX from the nanoparticles, release of DOX from the endosomal compartments, and the induced photothermal effects.

## 2 Results

### 2.1 Characterization of Functionalized Gold Nanoparticles

**Figure 1:**
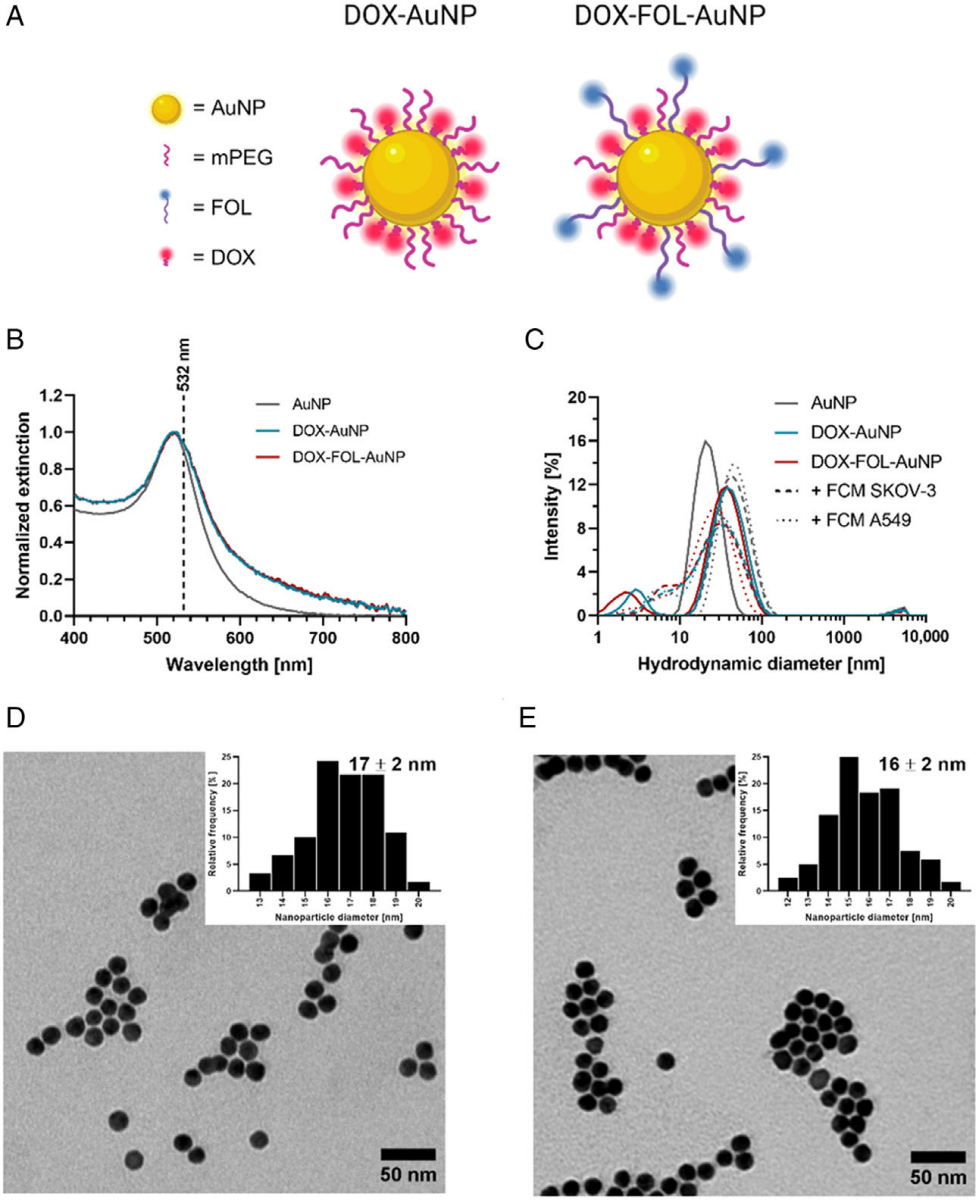
Characterization of DOX and DOX‐FOL‐coated AuNPs. A) Schematic overview of the DOX‐AuNP and DOX‐FOL‐AuNP formulations used in this study. B) Normalized UV/vis spectra of AuNPs (grey), DOX‐AuNPs (blue), and DOX‐FOL‐AuNPs (red). C) Intensity size distributions of AuNPs (grey), and the DOX‐AuNP formulations in HyClone Pure water (full line), and after 4 h incubation in FCM for SKOV‐3 cells (dashed) or A549 cells (dotted) for both DOX‐AuNPs (blue) and DOX‐FOL‐AuNPs (red). D,E) Representative TEM images of DOX‐AuNPs (D) and DOX‐FOL‐AuNPs (E). Scale bars = 50 nm. Size distribution and mean diameter ± standard deviation are displayed in the top‐right corner.

AuNPs were synthesized using a method adapted from Turkevich et al. (1953)^[^ ^51^ ^]^ and Li et al. (2011).^[^ ^52^ ^]^ In a multistep procedure, the AuNPs were subsequently covalently functionalized with FOL‐PEG_3.5kDa_‐SH, the releasable prodrug of DOX *lipoyl‐hydrazone‐*DOX, and mPEG_2kDa_‐SH (mPEG) to impart colloidal stability and stealth features.^[^ ^53^ ^]^ As a targeting control, DOX‐AuNPs were also synthesized (i.e., without FOL). This resulted in DOX‐AuNPs and DOX‐FOL‐AuNPs (**Figure** **1**A). These AuNP formulations were lyophilized using a suitable ratio and concentration of cryoprotectants, selected based on a dedicated screening study,^[^ ^54^ ^]^ to provide adequate storage stability. Upon resuspension, both DOX‐AuNPs and FOL‐DOX‐AuNPs had near‐identical UV/visible spectra (Figure 1B), with surface plasmon resonance at 520 nm, suggesting that the AuNP core had a diameter of ≈12–20 nm.^[^ ^55^ ^]^ Moreover, the slight broadening of the UV/visible spectra compared to the citrate‐capped AuNPs indicated successful coating. The spectra also indicated that the formulations were able to interact with the 532 nm laser light used in this study. Analysis by dynamic light scattering (DLS) revealed a Z‐average hydrodynamic diameter of 25.8 ± 0.7 nm for DOX‐AuNPs and 21.1 ± 0.3 nm for DOX‐FOL‐AuNPs, with a polydispersity index (PDI) of 0.485 ± 0.023 and 0.442 ± 0.006 and a zeta potential of −27.0 ± 5.0 and −20.9 ± 0.5 mV, respectively (Figure 1C and Table S1, Supporting Information). Even after 4 h of incubation in the full culture media (FCM) of SKOV‐3 and A549 cells, the hydrodynamic diameter and PDI remained unchanged (Figure 1C and Table S1, Supporting Information), demonstrating that these formulations do not aggregate within the required timeframe for the cell experiments in this study. Citrate‐capped AuNPs, however, did grow larger in size in the FCMs, again pointing toward successful functionalization of DOX‐AuNPs and DOX‐FOL‐AuNPs (Figure 1C and Table S1, Supporting Information). Transmission electron microscopy (TEM) confirmed that the nanoparticles had a near‐spherical shape (Figure 1D,E) with a diameter of *≈*16–17 nm, regardless of the functional moieties grafted on the surface.

### 2.2 Nanoparticle Conjugation Influences Nanoparticle Uptake and Clustering State in Endosomes

**Figure 2:**
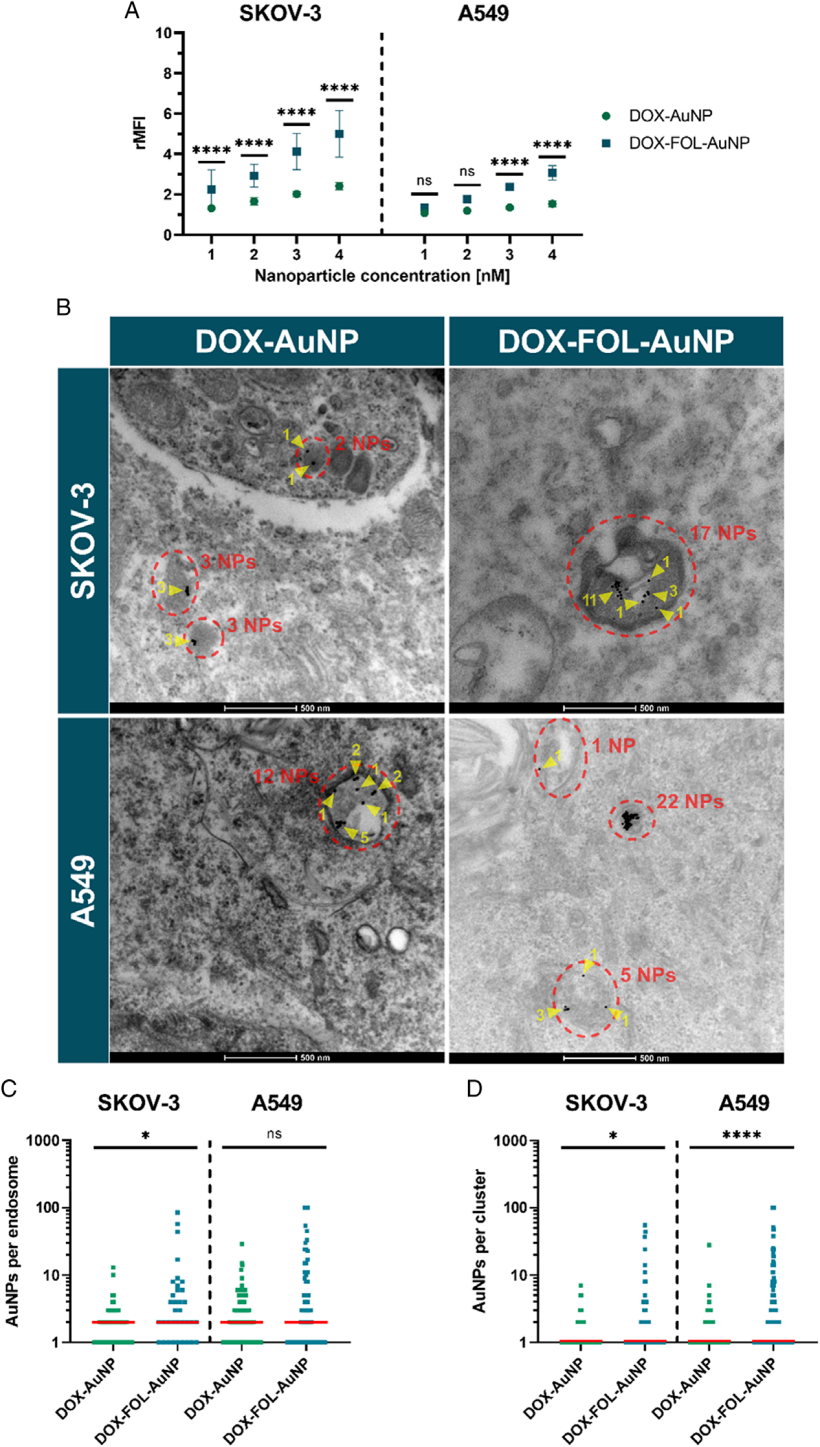
Quantification of the number of DOX‐AuNPs and DOX‐FOL‐AuNPs per cell and intracellular clustering state. A) Fluorescently labeled DOX‐AuNPs (green circles) and DOX‐FOL‐AuNPs (blue squares) were incubated with SKOV‐3 cells and A549 cells. Their number per cell was quantified by flow cytometry as rMFI. Data are represented by the mean ± standard deviation of three biological replicates, each consisting of three technical replicates. Two‐way analyses of variance (ANOVAs) with Tukey‐corrected multiple comparison tests were performed to determine statistically different results between DOX‐AuNP and DOX‐FOL‐AuNP rMFIs. B) Representative TEM images of SKOV‐3 and A549 cells incubated with DOX‐AuNPs and DOX‐FOL‐AuNPs. Red ellipses were drawn around individual endosomes containing nanoparticles, while yellow arrowheads indicate nanoparticle clusters within endosomes. Scale bars = 500 nm. C) Distribution of the number of DOX‐AuNPs and DOX‐FOL‐AuNPs per nanoparticle‐containing endosome in SKOV‐3 cells or A549 cells. Median is indicated by a red horizontal bar. D) Distribution of the number of DOX‐AuNPs and DOX‐FOL‐AuNPs per individual cluster in endosomes of SKOV‐3 cells and A549 cells. Median is indicated by a red horizontal bar. Kruskal–Wallis tests were used in (C) and (D) to determine statistically significant different distributions. *p < 0.05, ****p < 0.0001.

Next, we investigated the interaction of the nanoparticles with the FR‐expressing SKOV‐3 cell line,^[^ ^49^ ^]^ while the A549 cell line served as a non‐FR‐expressing control.^[^ ^50^ ^]^ Both cell lines are commonly used in the assessment of FR targeting.^[^ ^50^, ^56^, ^57^, ^58^, ^59^ ^]^ To do this, the DOX‐functionalized nanoparticles were fluorescently labeled with BDP 630/650, allowing quantification by flow cytometry after 4 h of incubation with nanoparticles. Specifically, the median fluorescence intensity relative to untreated cells (rMFI) was quantified as a measure of the number of nanoparticles per cell (**Figure** **2**A). Across the concentration range of 1–4 nM, FOL functionalization significantly increased the number of particles per SKOV‐3 cell. In A549 cells, FOL functionalization did not significantly increase the number of particles per cell at the lowest concentrations (1 and 2 nM). While a significantly higher number of DOX‐FOL‐AuNPs was also noted in A549 cells at 3 and 4 nM, it was not as much as observed in SKOV‐3 cells.

TEM confirmed successful nanoparticle uptake by cells after 4 h of incubation (Figure 2B). For all formulations and cell types, the particles were consistently found in endosomal vesicles, confirming that uptake occurs through endocytosis. We also quantified the number of AuNPs per endosome and their clustering state, as a previous study using siRNA‐loaded AuNPs has shown that this can influence their light‐triggered endosomal escape upon nanosecond laser irradiation.^[^ ^60^ ^]^ The number of DOX‐FOL‐AuNPs per endosome was significantly higher than that of DOX‐AuNPs in the SKOV‐3 cells, but not in A549 cells (Figure 2C). Furthermore, it was observed that within the endosomes, DOX‐FOL‐AuNPs tended to cluster more than DOX‐AuNPs (Figure 2D).

### 2.3 Vapor Nanobubbles Observed with Increasing Laser Fluence

**Figure 3:**
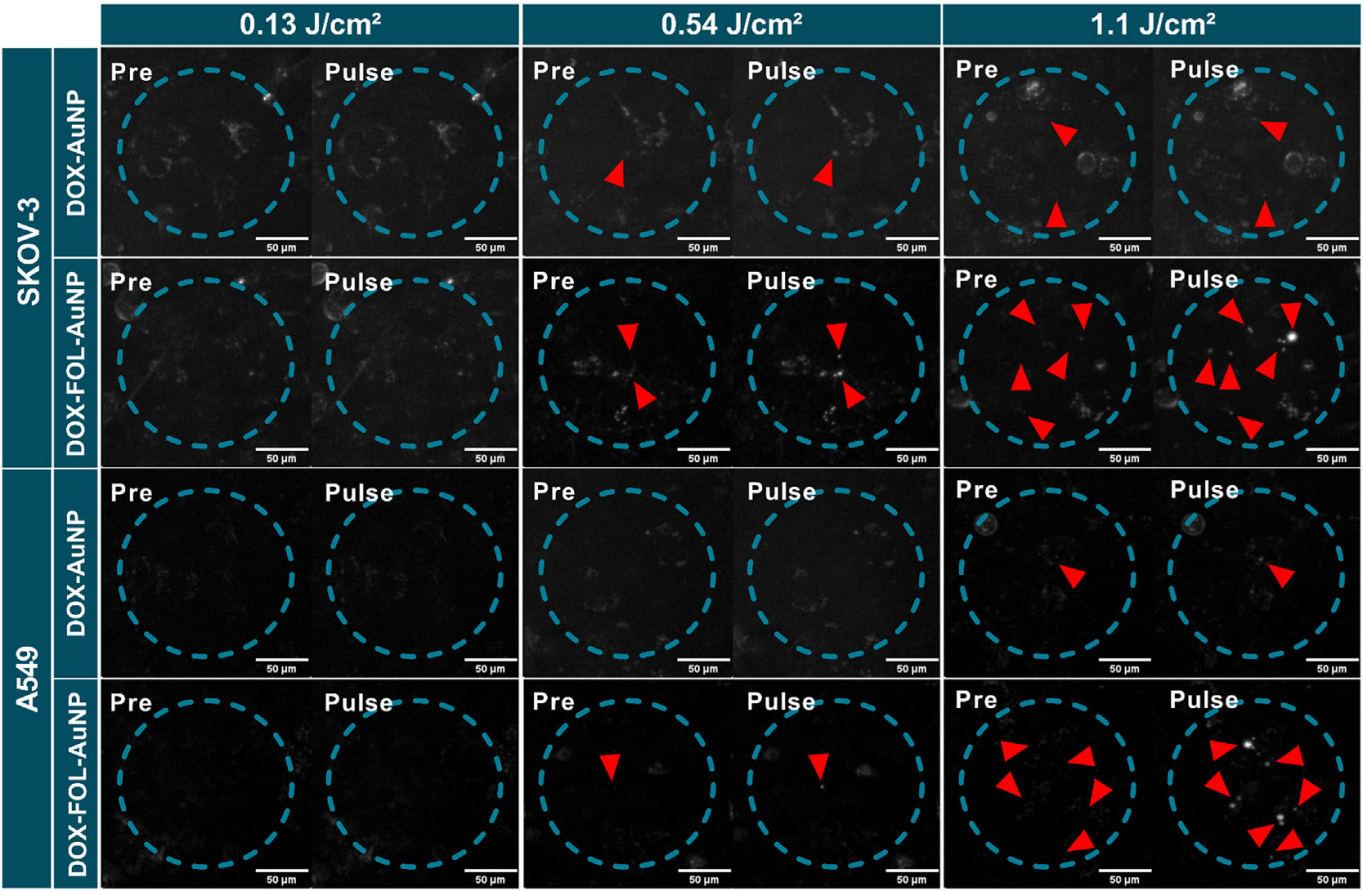
Visualization of VNB generation by DOX‐AuNPs and DOX‐FOL‐AuNPs per cell type. Dark‐field microscopy images to visualize VNB formation (red arrows) for increasing laser pulse fluences, where the irradiated zone is delineated with a dashed blue circle. SKOV‐3 and A549 cells were incubated with 4 nM DOX‐AuNPs or DOX‐FOL‐AuNPs for 4 h. Scale bars = 50 μm.

When endocytosed AuNPs are irradiated by nanosecond pulsed laser light, either heat or VNBs will emerge as the predominant photothermal mechanism depending on the applied laser pulse fluence.^[^ ^60^ ^]^ As the fluence increases, the AuNPs heat up more, eventually to the point where the surrounding liquid begins to evaporate. This results in the formation of expanding VNBs around the AuNPs, which collapse once the thermal energy from the absorbed laser pulse is exhausted. To investigate VNB formation from DOX‐AuNPs and DOX‐FOL‐AuNPs after being taken up by cells, we utilized dark‐field microscopy, which allows for the detection of these short‐lived VNBs as small bright bursts of scattered light.^[^ ^46^ ^]^ We tested three fluences, 0.13, 0.54, and 1.1 J cm^−2^. As expected, no VNBs could be seen when untreated cells were exposed to pulsed laser irradiation, even at the highest fluence of 1.1 J cm^−2^ (Figure S1, Supporting Information). Also, at the lowest fluence of 0.13 J cm^−2^ none of the AuNP formulations showed VNB formation in either of the cell lines (**Figure** **3**). At the intermediate laser pulse fluence of 0.54 J cm^−2^, an average of 1 ± 1 VNBs were observed in SKOV‐3 cells within the irradiation area for DOX‐AuNPs, while 3 ± 2 VNBs were observed for DOX‐FOL‐AuNPs. In A549 cells, there were no VNBs observed for DOX‐AuNPs, and only 1 ± 1 VNB for DOX‐FOL‐AuNPs. At 1.1 J cm^−2^ in SKOV‐3 cells, DOX‐AuNPs generated 6 ± 4 VNBs, while DOX‐FOL‐AuNPs generated 9 ± 1 VNBs. In A549 cells there were 0 ± 1 VNBs for DOX‐AuNPs and 6 ± 4 VNBs for DOX‐FOL‐AuNPs. These results are in line with the nanoparticle uptake data, as the enhanced uptake and clustering of the DOX‐FOL‐AuNPs (compared to DOX‐AuNPs) in both cell lines did indeed result in more VNBs upon laser treatment. Moreover, since SKOV‐3 cells were more prone to take up nanoparticles, they generated VNBs more readily at an intermediate laser pulse fluence. The observed bright bursts of light are truly caused by the scattering of the induced VNBs and not by the potential contribution of DOX fluorescence that may occur when exciting the molecule at 532 nm as evidenced by control experiments without using dark‐field illumination (Figure S2, Supporting Information).

### 2.4 DOX‐FOL‐AuNPs Induce Stronger Cytotoxicity Than DOX‐AuNPs in SKOV‐3 Cells Upon Pulsed Laser Irradiation

**Figure 4:**
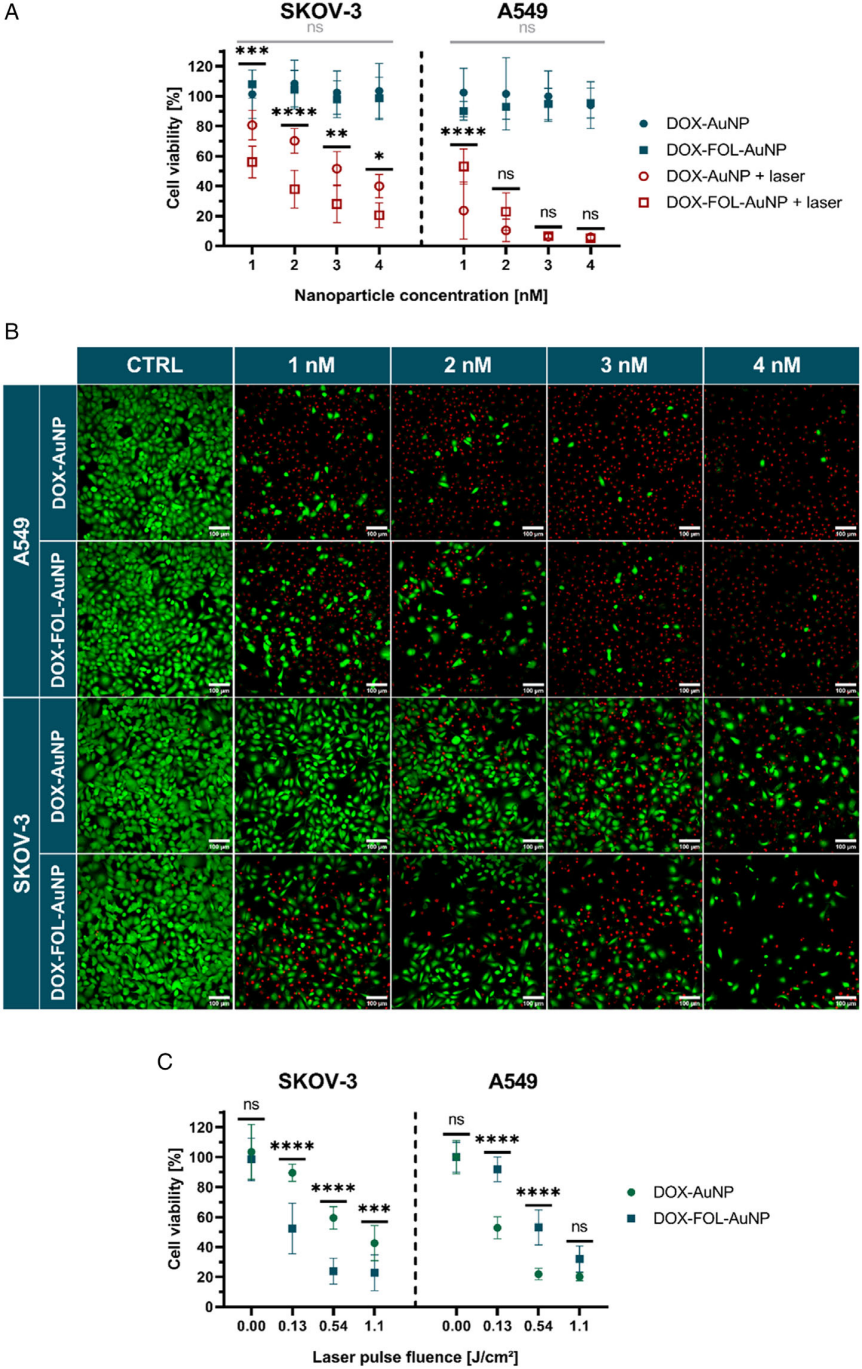
Cytotoxicity by DOX‐AuNPs and DOX‐FOL‐AuNPs upon pulsed laser irradiation. A) Effect of various concentrations of DOX‐AuNPs (circles) and DOX‐FOL‐AuNPs (squares) on relative cell viabilities––as measured by a CellTiter Glo assay 4 h post‐treatment––without (blue filled symbols) and with (red outlined symbols) 1.1 J cm^−2^ pulsed laser irradiation on SKOV‐3 and A549 cells. C) Representative live/dead confocal images of the tested conditions in (A). Cells incubated with 4 nM of the formulations represent the control to show no decrease in cell density (CTRL) if laser irradiation is absent. Viable cells were stained with calcein AM (green), while nuclei of dead cells were stained with TO‐PRO3 iodide (red). Scale bars = 100 μm. B) Cell viabilities for different laser pulse fluences and fixed nanoparticle concentrations (4 nM on SKOV‐3 and 2 nM on A549 cells). Two‐way ANOVAs with Tukey‐corrected multiple comparison tests were performed to determine statistically different results between formulations. In (A), the statistical significance of the difference between laser treated AuNP formulations is indicated in black, while it is indicated in gray for nonlaser‐treated AuNP formulations. Data are represented as the mean ± standard deviation of three biological replicates, each consisting of three technical replicates. *p < 0.05, **p < 0.01, ***p < 0.001, ****p < 0.0001.

Next, we evaluated the cytotoxicity of DOX‐AuNPs and DOX‐FOL‐AuNPs on SKOV‐3 and A549 cells using a CellTiter‐Glo assay. Without laser treatment, neither of the formulations showed any toxic effect at nanoparticle concentrations between 1 and 4 nM (**Figure** **4**A). However, when pulsed laser irradiation was applied at 1.1 J cm^−2^, a concentration‐dependent decrease in viability was observed (Figure 4A). DOX‐FOL‐AuNPs had a stronger effect than DOX‐AuNPs on SKOV‐3 cells, highlighting the advantage of increasing particle uptake by targeting the FR. Instead, targeting the FR did not increase the observed toxicity in A549 control cells, even at less effective conditions that did not induce virtually complete cell killing. Confocal images of cells stained with a combination of the pancellular viability dye calcein AM and TO‐PRO3 iodide––for live and dead cells, respectively––confirmed these trends (Figure 4B).

Next, we explored the influence of laser pulse fluence, while keeping the nanoparticle dose constant. On SKOV‐3 cells, a concentration of 4 nM was selected. However, based on the previous results, we selected a particle concentration of 2 nM for A549 cells since higher concentrations proved extremely toxic. The advantage of FOL functionalization was apparent on SKOV‐3 cells for all three fluences (0.13, 0.54, 1.1 J cm^−2^), with enhanced killing for DOX‐FOL‐AuNPs compared to DOX‐AuNPs (Figure 4C). However, there was no enhanced cell killing for DOX‐FOL‐AuNPs in comparison to DOX‐AuNPs on A549 cells. Interestingly, cell killing increased with laser fluence in all cases.

Given that apoptosis is the main induced regulated cell death pathway by DOX^[^ ^61^ ^]^ and a generally well‐established form of regulated cell death for such combination systems,^[^ ^62^ ^]^ we performed a Caspase‐Glo assay to assess caspase 3/7 activity 4 h post‐treatment. As shown in Figure S3A,B, Supporting Information, a decrease in relative caspase 3/7 activity was noted when using identical conditions as those in Figure 4. This observation is a consequence of a loss of cells due to cytotoxicity (Figure 4). Therefore, we normalized the caspase activities to the remaining living cells, finding that the normalized caspase 3/7 activity increased as conditions became more stringent (Figure S3C,D, Supporting Information). This suggests that apoptosis occurs in cells that do not die instantaneously after treatment. Interestingly, this effect was more pronounced for DOX‐FOL‐AuNPs with respect to DOX‐AuNPs in SKOV‐3 cells, both when varying the nanoparticle concentration (Figure S3C, Supporting Information) and laser pulse fluence (Figure S3D). This difference was not as apparent when considering the effects in A549 cells (Figure S3C,D, Supporting Information). Overall, the induced cytotoxicity––whether related to apoptotic cell death or not––could be because of photothermal effects (heat and VNB‐formation), or because higher fluences enhance DOX release from endosomes. Both aspects will be further investigated in Section 2.5 and 2.6, respectively.

### 2.5 Cytotoxicity Is Induced by a Combination of Photothermal Effects and Light‐Triggered Release of Doxorubicin

To evaluate the extent to which photothermal effects contribute to the observed cytotoxicity, similar AuNP formulations were synthesized but without DOX, resulting in mPEG‐AuNPs and FOL‐AuNPs (Figure S4A, Supporting Information). mPEG**‐**AuNPs and FOL‐AuNPs had similar physicochemical characteristics as their DOX‐functionalized counterparts, including their extinction spectrum (Figure S4B, Supporting Information), particle size (Figure S4C and Table S2, Supporting Information), morphology (Figure S4D,E, Supporting Information), and zeta potential (Table S2, Supporting Information). Intracellular uptake was similar for mPEG‐AuNPs (Figure S5, Supporting Information) as for DOX‐AuNPs in both cell lines (Section 2.2). Surprisingly, however, FOL‐AuNPs (Figure S5, Supporting Information) did not show the same levels of uptake in either cell line when compared to DOX‐FOL‐AuNPs (Section 2.2). Therefore, we continued comparing mPEG‐AuNPs and DOX‐AuNPs only, as it is important that the level of uptake is the same for the formulations with and without DOX if we want to determine the contribution of photothermal effects to the observed toxicity.

**Figure 5:**
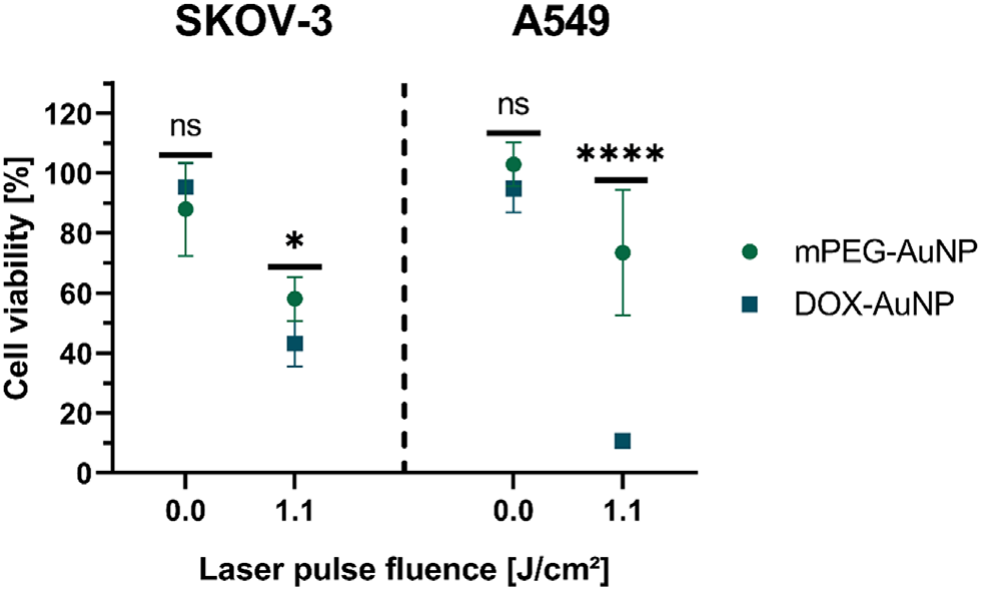
Contribution of photothermal effects to the perceived toxicity upon pulsed laser treatment, as determined using AuNP formulations without DOX functionalization. Comparison of cytotoxicity induced by mPEG‐AuNPs (green circles) and DOX‐AuNPs (blue squares) on relative cell viabilities—as measured by a CellTiter Glo assay 4 h post‐treatment—without and with 1.1 J cm^−2^ pulsed laser irradiation on SKOV‐3 and A549 cells. A one‐way ANOVA with a Sidak‐corrected multiple comparison test was used to determine statistically different viabilities between the two formulations. Data are represented by the mean ± standard deviation of three biological replicates, each consisting of three technical replicates. *p < 0.05, ****p < 0.0001.

We incubated both cell lines with mPEG‐AuNPs and DOX‐AuNPs at the highest concentration tested (4 nM) and evaluated cell viability with and without laser treatment at the highest laser pulse fluence (1.1 J cm^−2^) (**Figure** **5**B). As expected from the results in Section 2.4, limited toxicity was observed without laser irradiation. For mPEG‐AuNPs, the viability decreased to 58 ± 7% and 74 ± 21% upon laser treatment of SKOV‐3 and A549 cells, respectively. A stronger decrease in viability was measured when using DOX‐AuNPs, in which case laser treatment resulted in a viability of 43 ± 8% for SKOV‐3 cells and 11 ± 2% for A549 cells. Therefore, while photothermal effects did trigger a noticeable toxicity on these cells, this was to a statistically significant lesser extent than when DOX‐functionalized particles were used, demonstrating that both chemotoxicity by DOX and photothermal mechanisms play a role in the observed killing effect of DOX‐loaded AuNPs stimulated by nanosecond pulsed laser light.

### 2.6 Laser Irradiation of Doxorubicin‐Coated Nanoparticles Releases Doxorubicin and Prompts Endosomal Escape

**Figure 6:**
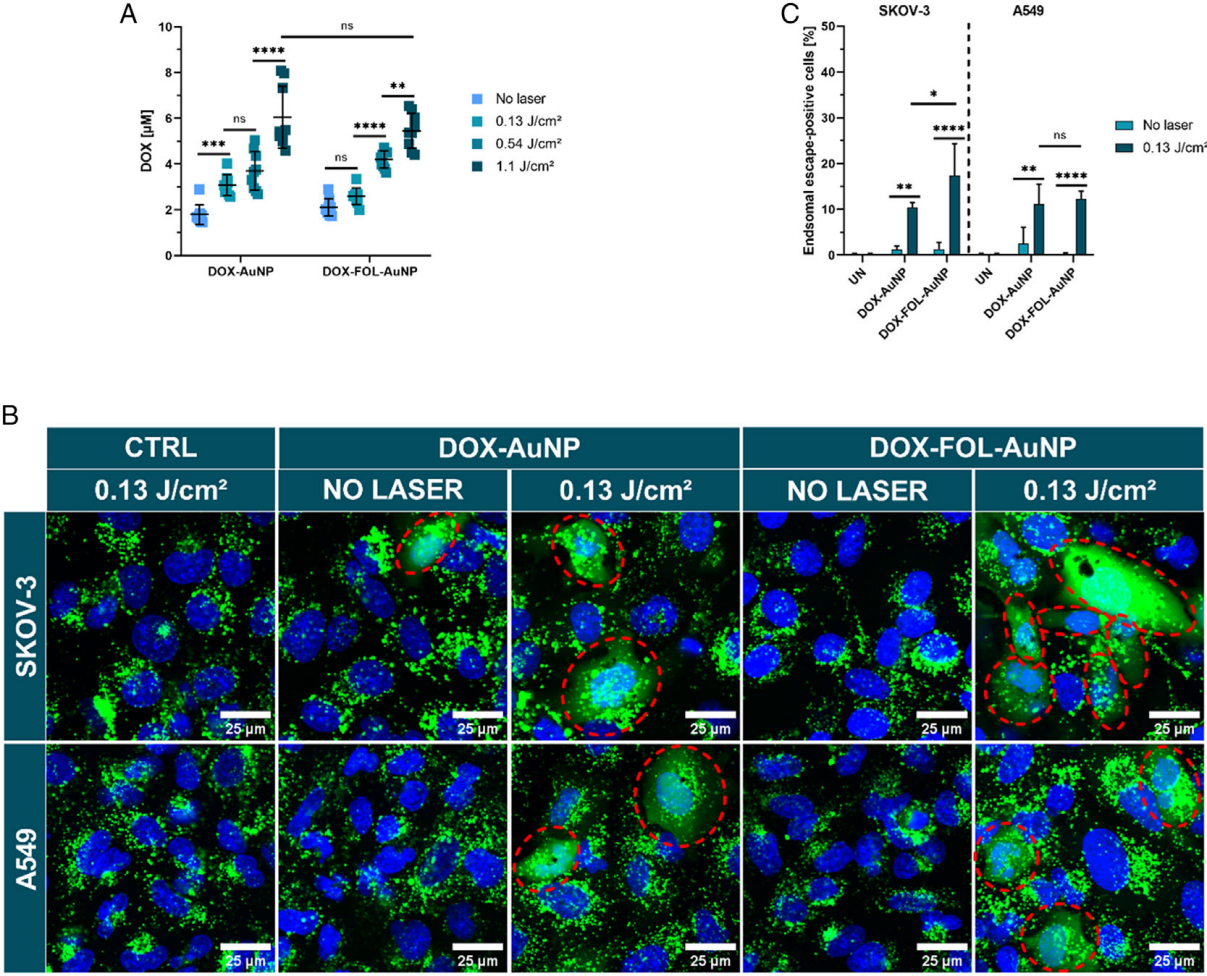
Light‐triggered release of DOX and laser‐induced endosomal escape in SKOV‐3 and A549 cells incubated with DOX‐AuNPs and DOX‐FOL‐AuNPs. A) Release of DOX was determined using fluorimetry by irradiation of DOX‐AuNPs and DOX‐FOL‐AuNPs with the various laser pulse fluences used in this study: 0.13 J cm^−2^ (light blue), 0.54 J cm^−2^ (darker blue), and 1.1 J cm^−2^ (darkest blue). B) Representative confocal microscopy images of the calcein release assay for investigating endosomal escape. Endosomal escape is apparent from a uniform staining of the cell by calcein (green), while a punctuate pattern is observed in the absence of endosomal escape. Nuclei of cells were stained with Hoechst 33 342 (blue). Dashed red ellipses indicate successful endosomal escape events. Scale bars = 50 μm. Endosomal escape was investigated using a calcein release assay in SKOV‐3 and A549 cells incubated with 2 nM DOX‐(FOL‐)AuNPs and stimulated with 0.13 J cm^−2^ laser irradiation. C) The percentage of cells showing endosomal escape, which was determined from analyzing >500 cells per condition. In (A) and (C), two‐way ANOVAs with Tukey‐corrected multiple comparison tests were performed to determine statistically different results within groups and Sidak‐corrected multiple comparisons tests to test statistically different results between groups. Data are represented by the mean ± standard deviation of (A) three functionalized AuNP batches and (C) three biological replicates, each consisting of three technical replicates. *p < 0.05, **p < 0.01, ***p < 0.001, ****p < 0.0001.

Next, we wanted to confirm light‐triggered release of DOX from the AuNP surface upon nanosecond laser pulse stimulation. Suspensions of DOX‐AuNPs and DOX‐FOL‐AuNPs were exposed to nanosecond pulsed laser light (one pulse per location in the sample), and the released DOX was separated from the nanoparticles by centrifugation and quantified in the supernatant using fluorimetry (Figure S6A, Supporting Information) following the establishment of a calibration curve (Figure S6B, Supporting Information). In the absence of laser irradiation, 1.79 ± 0.43 and 2.10 ± 0.37 μM free DOX was found in the DOX‐AuNP and DOX‐FOL‐AuNP samples, respectively (**Figure** **6**A). Gradually increasing amounts of free DOX were observed with increasing laser pulse fluences, reaching 6.0 ± 1.4 and 5.46 ± 0.77 μM at the highest fluence of 1.1 J cm^−2^ for the DOX‐AuNPs and DOX‐FOL‐AuNPs, respectively. These results show that light‐triggered release of DOX scales with the applied laser pulse fluence.

Knowing that the nanoparticles are entrapped in endosomal vesicles in the cells, we next assessed the ability of the laser‐irradiated nanoparticle formulations to disrupt the endosomal membrane, which should facilitate DOX release into the cytosol. Endosomal rupture was investigated using a calcein release assay and confocal microscopy.^[^ ^63^ ^]^ As long as the endosomes remain intact, calcein will be trapped inside of them, resulting in a punctuate quenched fluorescent pattern. But when the endosomal wall is permeabilized, calcein will escape into the cytosol, resulting in a brighter homogeneous staining of the cell. SKOV‐3 cells or A549 cells that were incubated with 2 nM DOX‐AuNPs or DOX‐FOL‐AuNPs did not show any endosomal escape in the absence of laser stimulation (Figure 6B). Similarly, when a laser pulse fluence of 0.13 J cm^−2^ was applied in the absence of nanoparticles, no noticeable endosomal escape was observed either. However, when laser irradiation and nanoparticles were combined, clear endosomal escape occurred, as indicated by the many uniformly stained cells. Quantification of confocal images revealed that endosomal escape occurred in 10 ± 1% of SKOV‐3 cells treated with DOX‐AuNPs, which increased to 17 ± 7% for DOX‐FOL‐AuNPs (Figure 6C). In A549 cells, the percentage remained constant at about 12% for both DOX‐AuNPs and DOX‐FOL‐AuNPs. When higher laser fluences and nanoparticle concentrations were tested to induce more endosomal escape, the high cytotoxicity under these conditions resulted in massive cell loss, particularly in cells where endosomal escape occurred, making accurate quantifications impossible.

### 2.7 Laser Activation of DOX‐AuNPs Allows Killing Cells with High Spatial Accuracy

**Figure 7:**
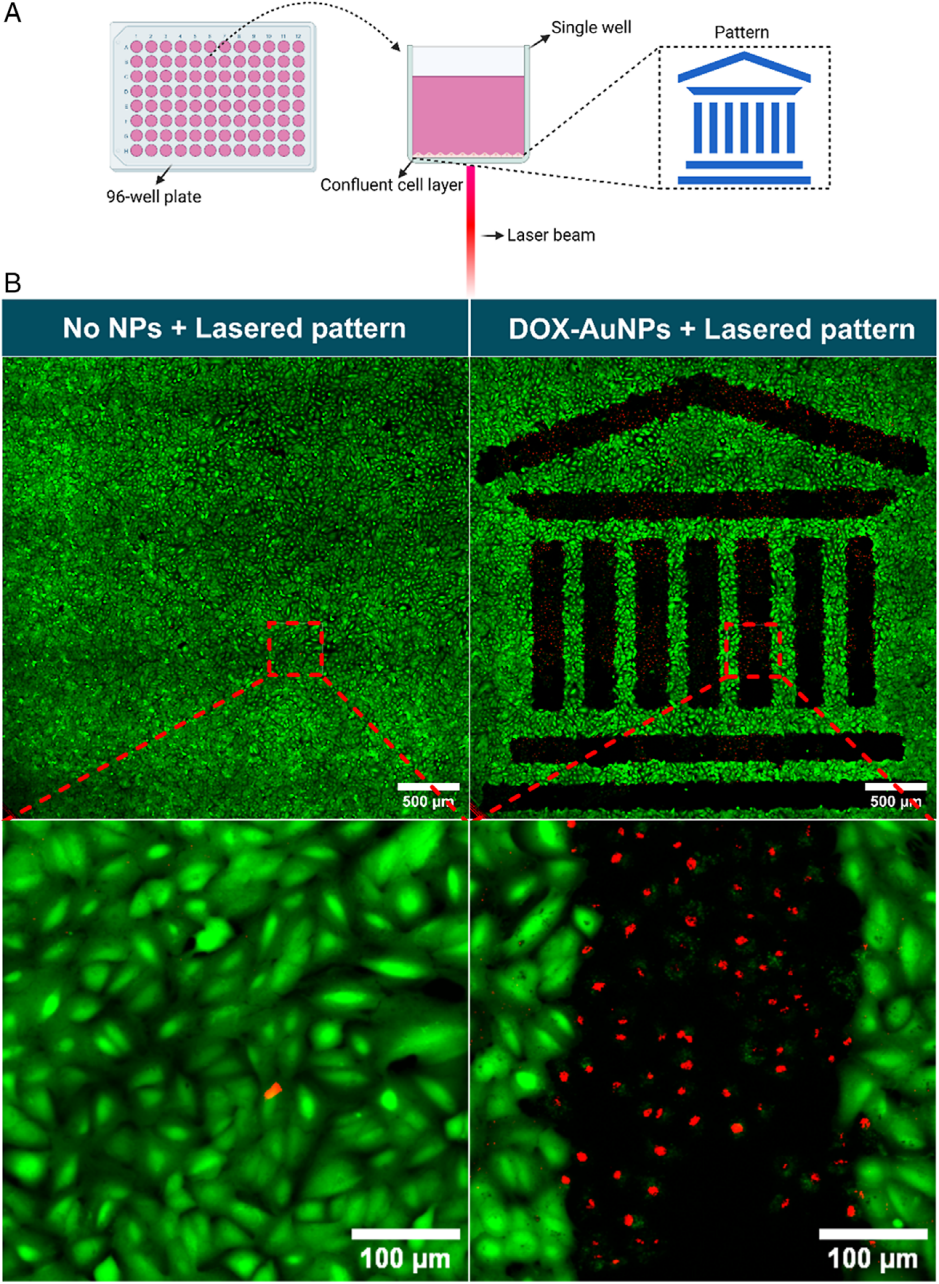
Spatially selective induced cytotoxicity by DOX‐AuNPs upon pulsed laser irradiation. A) Schematic overview of spatial‐selective laser treatment. B) A549 cells without and with DOX‐AUNPs irradiated at 1.1 J cm^−2^ according to a pattern resembling the Ghent University logo. Viable cells were stained with calcein AM (green), while nuclei of dead cells were stained with TO‐PRO3 iodide (red). Scale bars = 500 μm (original) and 100 μm (zoomed).

An important advantage of using nanosecond pulsed laser irradiation is the possibility to carefully control the area over which the treatment is applied, thus reducing off‐target effects. To illustrate the level of spatial control, A549 cells, which had proven to be the most sensitive cells, were incubated for 4 h with 4 nM DOX‐AuNPs and irradiated at a laser pulse fluence of 1.1 J cm^−2^ in a pattern resembling the Ghent University logo (**Figure** **7**A). Live cells were stained using the pancellular calcein AM viability dye, while dead cells were stained using TO‐PRO3 iodide. In the absence of DOX‐AuNPs, the laser irradiation did not affect cell viability (Figure 7B). However, following incubation with DOX‐AuNPs, cells were selectively killed in the irradiated areas, inducing complete ablation, whereas cells remained unaffected in the nonirradiated areas (Figure 7B). More images of spatially resolved killing using DOX‐AuNPs and DOX‐FOL‐AuNPs in A549 and SKOV‐3 are shown in Figure S7, Supporting Information. Together this illustrates the possibility of nanosecond laser stimulation to induce highly localized cytotoxicity.

## 3 Discussion

Among the recent efforts to develop more efficacious and safer anticancer therapies, nanoparticle drug carriers tailored for targeted and/or controlled release of therapeutic drugs have gained considerable interest.^[^ ^18^ ^]^ One avenue involves the use of chemotherapeutic‐loaded photothermal nanoparticles to achieve spatiotemporally controlled release and cell killing upon laser irradiation.^[^ ^38^, ^63^, ^64^, ^65^ ^]^

Here, AuNPs were functionalized with releasable DOX as a commonly used chemotherapeutic.^[^ ^65^ ^]^ In addition, the AuNPs were functionalized with FOL as a targeting ligand, considering that a significant number of tumor cells overexpresses the FR.^[^ ^22^ ^]^ The AuNP surface was further saturated with mPEG, which is frequently used as a moiety to increase stability, provide stealth features, and prolong nanoparticle circulation times.^[^ ^53^ ^]^ UV/vis spectrophotometry indicated that both formulations had a near‐identical spectrophotometric profile. DLS measurements showed that both formulations also had a similar zeta potential and size, even in full cell culture medium. TEM images did not show a corona of functional moieties on the AuNP surface, as visualized in prior work,^[^ ^66^ ^]^ due to a modified sample preparation. Yet, it proved the photothermal AuNP core to be identical across formulations. Considering these results, a direct comparison of laser‐induced effects on cells between the two AuNP formulations was possible.

SKOV‐3 cells were included as a cell line that expresses the FR,^[^ ^49^ ^]^ while A549 cells that do not express this receptor served as a control,^[^ ^50^ ^]^ and both have been used in FR‐targeting studies.^[^ ^50^, ^56^, ^57^, ^58^, ^59^ ^]^ Following incubation with SKOV‐3 cells, the number of DOX‐FOL‐AuNPs per cell was markedly higher than for DOX‐AuNPs, confirming improved affinity due to FOL functionalization. This was not the case for A549 cells when using particle concentrations of 1 and 2 nM. However, at higher concentrations (3 and 4 nM), more DOX‐FOL‐AuNPs were found in A549 cells as well when compared to DOX‐AuNPs, though the overall particle load per cell was lower with respect to the SKOV‐3 cells incubated with equivalent concentrations of the DOX‐FOL‐AuNPs. Although the reason remains unclear, it may be that FOL functionalization increases nonspecific interactions with the cell membrane due to a low affinity of FOL for phospholipids^[^ ^67^, ^68^, ^69^ ^]^ and/or to variations in cell membrane composition.^[^ ^70^, ^71^ ^]^ Note that this does not necessarily mean that the particles are internalized by the cells, considering that uptake was assessed by flow cytometry, which does not distinguish between extra‐ and intracellularly bound particles.

Therefore, the internalized AuNP formulations were analyzed. TEM revealed no discernable difference between the number of internalized DOX‐AuNPs and DOX‐FOL‐AuNPs in A549 cells. In contrast, more DOX‐FOL‐AuNPs were internalized per endosome than DOX‐AuNPs in SKOV‐3 cells, thus implying that the FR on these cells was successfully targeted. Yet, in both cell lines, the DOX‐FOL‐AuNPs tended to cluster more within the endosomes in comparison to DOX‐AuNPs. FOL functionalization may lower the antiaggregating properties of mPEG under acidifying conditions as encountered during endosomal maturation,^[^ ^72^, ^73^ ^]^ which can affect nanoparticle–nanoparticle interactions.^[^ ^74^, ^75^ ^]^

In this study we tested the use of a nanosecond pulsed laser irradiation to activate the DOX‐loaded AuNPs. Nanosecond pulsed lasers reduce potential damage to surrounding healthy cells by efficiently inducing effective photothermal effects on short timescales, requiring shorter treatment times than CW laser irradiation and thus being inherently more spatially confined (i.e., very limited heat diffusion). The two main photothermal effects that are expected to be elicited are heat generation or VNB formation above a certain threshold of the laser pulse fluence. Therefore, we investigated VNB formation for a low‐ (0.13 J cm^−2^), intermediate‐(0.54 J cm^−2^), and high‐(1.1 J cm^−2^) laser pulse fluence. Overall, VNBs were more readily observed in SKOV‐3 cells at the intermediate laser pulse fluence (0.54 J cm^−2^) compared to A549 cells. Similarly, FOL functionalization led to more VNB formation in SKOV‐3 cells. While these findings correlate with the particle update data, clustering of particles in the endosomes may also play a role in this observation, considering that DOX‐FOL‐AuNPs clustered more than DOX‐AuNPs in both cell lines. It has been reported before that AuNP clustering is beneficial for generating VNBs.^[^ ^60^, ^76^ ^]^ Overall, at higher laser pulse fluences, VNBs contribute to the photothermal effects besides heat generation.

Neither of both DOX‐containing particle formulations induced acute toxicity in the absence of nanosecond pulsed laser irradiation. However, when irradiating the cells at a laser fluence of 1.1 J cm^−2^, a concentration‐dependent cell killing was observed with a near‐complete eradication of the cells at the highest concentrations tested. Toxicity was higher for DOX‐FOL‐AuNPs in SKOV‐3 cells compared to DOX‐AuNPs, which was not the case for A549 cells, thus confirming the contribution of FR targeting in SKOV‐3 cells. Using similar nanoparticles without DOX, we established that cytotoxicity is induced by a combination of photothermal effects and chemotoxicity by DOX. In absolute terms, cytotoxicity was higher in A549 cells, which may be the result of a higher sensitivity to DOX,^[^ ^77^, ^78^, ^79^, ^80^ ^]^ general differences in (the extent of) triggered cell death pathways due to distinct cell (pheno)types,^[^ ^81^, ^82^ ^]^ or both. There were some indications of apoptosis occurring in a fraction of the laser‐treated cells, which can possibly be ascribed to DOX‐induced chemotoxicity, photothermal effects, or both.^[^ ^61^, ^62^ ^]^ Nonetheless, the apoptotic activity did not explain all observed toxicities. Future, more extensive investigations can elucidate the specific induced cell death pathways and uncover the proportion and additive or synergistic nature of the contributing effects (i.e., photothermal effects and DOX‐induced chemotoxicity).

We also investigated whether DOX is released upon pulsed laser irradiation. DOX release was confirmed from both DOX‐FOL‐AuNPs and DOX‐AuNPs in a fluence‐dependent manner. Disruption of the endosomal membrane proved already possible at the lowest laser pulse fluence, facilitating released DOX to enter the cytosol and exert its pleiotropic cytotoxic functions.^[^ ^61^, ^83^ ^]^ DOX‐FOL‐AuNPs induced endosomal escape in more SKOV‐3 cells compared to DOX‐AuNPs, which was not the case for A549 cells. Again, this supports the role of FOL as a successful targeting moiety.

The possibility to induce cell killing with high spatial precision was illustrated by irradiating A549 cells incubated with DOX‐AuNPs according to a predefined pattern. The sharp boundaries between irradiated (killed) and unirradiated (healthy) cells indicated that cell killing with nanosecond pulsed laser irradiation can be strictly confined to the irradiated cells without harming neighboring cells.

With an eye on the translational potential, future work should focus on the spatial‐selective cytotoxicity and long‐term effects in more complex mixed cultures and tissue mimics containing both malignant and normal cells. Investigating the safety profile of the proposed treatment using monocultures of normal healthy cells is also of interest. When considering future in vivo experiments, both superficial and deep cancers can be targets of these light‐sensitive drug delivery systems, even in combination with irradiation in the visible range (such as the 532 nm laser used here).^[^ ^84^, ^85^ ^]^ This is exemplified by intratumorally injected spherical AuNPs for phototherapy of superficial oral mucosa cancers,^[^ ^86^ ^]^ whereas optical fibers for the treatment of bladder cancer have also been successfully used to provide the laser stimulation.^[^ ^87^, ^88^ ^]^ The combination with surgical interventions to reach deeper cancers—such as liver, lung, eye and bone cancers—^[^ ^89^, ^90^, ^91^, ^92^ ^]^ is also of interest. In case deeper light penetration is required, spherical AuNPs can be exchanged for other AuNP morphologies which can be excited by NIR laser light, such as gold nanorods, gold nanocages, gold nanoshells, and gold nanorings, among others.^[^ ^93^, ^94^ ^]^ NIR excitation can enhance the penetration depth of light stimulation in, for example, solid tumors.^[^ ^95^, ^96^ ^]^

## 4 Conclusion

In this study, we have demonstrated that FOL functionalization leads to enhanced endocytic uptake of DOX‐loaded AuNPs in FR‐expressing SKOV‐3 cells. Stimulation with nanosecond laser pulses leads to release of DOX from the nanoparticles and permeabilization of the endosomal membranes, resulting in cell death by a combination of photothermal effects and chemotoxicity. Cell death can be precisely limited to the irradiated cells, leaving surrounding cells unharmed. Together, this study shows the promise of nanosecond pulsed laser irradiation in combination with DOX‐FOL‐AuNPs to kill FR‐expressing cancer cells with high specificity.

## 5 Experimental Section

5.1

5.1.1

##### Materials

FOL, sodium citrate tribasic dihydrate, tetrachloroauric(III) acid trihydrate, iodine, potassium iodide, barium chloride, N‐hydroxysuccinimide (NHS), N, N′‐dicyclohexylcarbodiimide (DCC), triethylamine, α‐lipoic acid, dimethylsulfoxide anhydrous (DMSO), chloroform, dichloromethane (DCM), diethyl ether (Et_2_O), Sephadex G‐25 superfine and Sephadex LH 20 gel filtration resins were purchased from Sigma‐Aldrich (St. Louis, MO, USA). DOX hydrochloride was purchased from LC Laboratories (Woburn, MA, USA). BDP 630/650 X NHS ester was obtained from Lumiprobe (Hannover, Germany). NH_2_‐PEG_2kDa_‐SH and NH_2_‐PEG_3.5kDa_‐SH were purchased from JenKemTechnology (Plano, TX, USA). mPEG_2kDa_‐SH was acquired from Iris Biotech GmbH (Marktredwitz, Germany).

##### Synthesis of Functional Components

**Figure 8:**
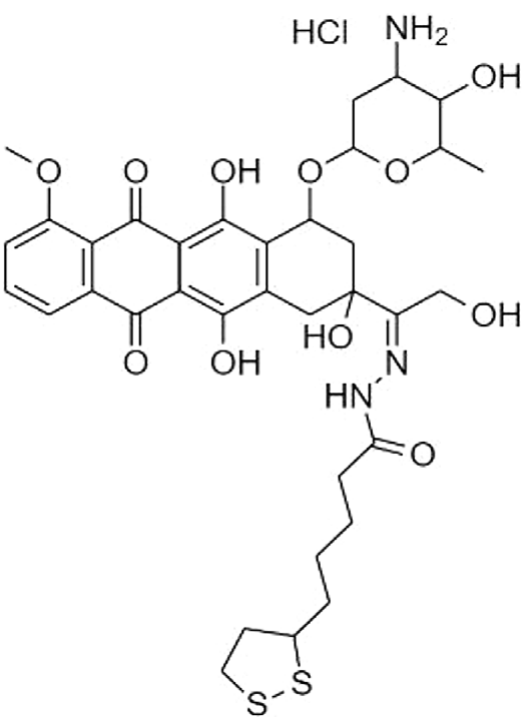
Chemical structure of lipoyl‐hydrazone‐DOX.

Synthesis and characterization of the targeting agent FOL‐PEG_3.5kDa_‐SH (FOL‐PEG) and the fluorescent probe BDP‐PEG_2kDa_‐SH (BDP‐PEG) were performed according to the literature.^[^ ^66^ ^]^ Briefly, FOL was activated to its N‐hydroxy succinimidyl ester using DCC and reacted with NH_2_‐PEG_3.5kDa_‐SH in DMSO under anhydrous conditions in the presence of triethylamine to yield the conjugate FOL‐PEG_3.5kDa_‐SH that underwent purification by size exclusion chromatography. BDP 630/650 X NHS was reacted in anhydrous DMSO with NH_2_‐PEG_2kDa_‐SH in the presence of triethylamine to provide BDP‐PEG_2kDa_‐SH that was purified by size exclusion chromatography. The releasable prodrug lipoyl‐hydrazone‐DOX (**Figure** **8**) was synthesized by the conversion of lipoic acid into its lipoyl‐hydrazide first and conjugation to the ketone group of DOX. The derivatives were characterized by ^1^H‐NMR, mass, and UV/vis spectroscopy (S. Salmaso et al., manuscript under submission).

##### Gold Nanoparticle Synthesis and Functionalization

AuNPs were generated by reduction of HAuCl_4_ with trisodium citrate using the Turkevich method with slight modifications.^[^ ^51^, ^52^ ^]^ The concentration of the AuNPs in the samples was assessed according to the procedure reported in the literature and our previous studies.^[^ ^66^, ^97^ ^]^ Fully decorated AuNPs (DOX‐FOL‐AuNPS) were obtained by freshly synthesized AuNP functionalization with targeting FOL‐PEG, fluorescent BDP‐PEG (where applicable), lipoyl‐hydrazone‐DOX, and stabilizing mPEG_2kDa_‐SH, according to a multistep protocol. A volume of 10 mL of 10 nM AuNPs in milliQ water was sequentially incubated for 3 h with each of the following mixture molar ratios: 1) 50:5:1 FOL‐PEG/mPEG_2kDa_‐SH/AuNPs, 2) 20:80:1 BDP‐PEG/mPEG_2kDa_‐SH/AuNPs, 3) 1000:1 lipoyl‐hydrazone‐DOX/AuNPs, and 4) 1000: mPEG_2kDa_‐SH/AuNPs. FOL‐PEG free particles (control particles: DOX‐AuNPs) were generated by replacing FOL‐PEG with mPEG_3.5kDa_‐SH. Particles without the DOX derivative (FOL‐AuNPs and mPEG‐AuNPs) were generated with the above reported procedure by omitting the aforementioned third incubation step. The conjugation yield of each functional component was derived indirectly from quantification of the nonconjugated moieties. After each functionalization step, particle mixtures were centrifuged at 13.500 RCF and FOL‐PEG in the supernatant was spectrophotometrically assessed at 363 nm, BDP‐PEG and lipoyl‐hydrazon‐DOX were spectrofluorimetrically assessed (*ʎ*_ex_ = 628 nm/*ʎ*_em_ = 642 nm and *ʎ*_ex_ = 488 nm/*ʎ*_em_ = 590 nm, respectively) and mPEG_2kDa_‐SH was quantified by iodine assay.^[^ ^98^ ^]^ Under the experimental conditions, complete conjugation of FOL‐PEG and BDP‐PEG was achieved, while the conjugation efficiency of the DOX prodrug was 91%.

##### Lyophilization and Reconstitution of AuNP Formulations

All AuNP formulations were lyophilized for storage stability by first mixing equal volumes of AuNPs, and a 1% w/v solution of 1:1 w w^−1^ polyvinylpyrrolidone 40 kDa (PVP) (Sigma‐Aldrich, St. Louis, MO, USA)/Tween 20 (Croda International PLC, Snaith, UK) mixture as cryoprotectants in milliQ‐water, which prevailed as most adequate choice in a dedicated screening study.^[^ ^54^ ^]^ The final cryoprotectant concentration was 0.5% w/v. The mixture was then submerged in liquid nitrogen for 2 min and freeze dried overnight (Hetossic lyophilizer, HETO Lab Equipment, Birkerod, Denmark). Reconstitution of the formulations was achieved by resuspending the AuNPs in Dulbecco's phosphate‐buffered saline without Ca^2+^ and Mg^2+^ [DPBS(−/−), Biowest, Nuaillé, France].^[^ ^99^ ^]^ Two washing steps were performed to remove the cryoprotectants and prevent potential cytotoxicity. The formulations were centrifuged for 30 min at 13.500 RCF and 4 °C, after which the supernatant was removed and fresh DBPS(−/−) was added to resuspend the AuNPs.

##### Physicochemical Characterization

DLS (Zetasizer Nano ZS, Malvern Instruments Co., Malvern, Worcestershire, UK) was used to measure the hydrodynamic diameters, while the zeta potential was measured using the electrophoretic light scattering capabilities of this instrument. UV/vis spectrophotometric analysis was performed with a Nanodrop 2000c spectrophotometer (Thermofisher, Rockford, IL, USA). Both UV/Vis and DLS measurements were performed on samples diluted sixfold in either HyClone pure water or supplemented cell culture medium (Section 5). TEM images yielded additional information regarding the AuNP formulations’ diameters and morphological characteristics. Here for, the AuNPs were blotted onto EM grids, stained by a negative staining and evaluated in a TEM, Tecnai G2 Spirit BioTWIN (Thermo Fisher Scientific, FEI Europe BV, Eindhoven, The Netherlands) at 120 kV.

##### Cell Culture

A549 (American Type Culture Collection, ATCC‐CCL‐185) human lung epithelial‐like cells were cultured in DMEM medium + GlutaMAX‐I (Gibco, Grand Island, NY, USA), and the FR‐positive SKOV3 (American Type Culture Collection, ATCC‐HTB‐77) human ovarian epithelial‐like cells were cultured in RPMI‐1640 + glutamine + absence of FOL (Gibco, Grand Island, NY, USA). Both culture media were supplemented with 10% fetal bovine serum (Biowest, Nuaillé, France) and 100 U ml^−1^ Penicillin/Streptomycin (Gibco, Grand Island, NY, USA). Both cell lines were passaged, with passage numbers being kept under 30, upon reaching confluency every 2 or 3 days, followed by storage in a humidified incubator (37 °C, 5% CO_2_).

##### Treatment of Cells with AuNP Formulations and Subsequent Laser Irradiation

24 h prior to performing an experiment, cells, regardless of the cell line, were seeded at a density of 25.000 cell/well in a flat‐bottomed 96‐well plate and incubated in an incubator (37 °C, 5% CO_2_). Next, the cells were washed once with DPBS(−/−) and AuNPs—dilutions prepared in full culture medium—were added to the wells. To promote initial AuNP–cell contact, the plate was centrifuged immediately after for 10 s in a plate centrifuge (Eppendorf, Hamburg, Germany) until 1300 RCF was reached. This was followed by an incubation step of 4 h (37 °C, 5% CO_2_) to allow cellular uptake of the nanoparticles. Unbound AuNPs were removed by washing the cells with DPBS(−/−), and fresh culture medium was added to each well. Where required, wells were treated with laser irradiation. Laser treatment was performed using an in‐house developed optical setup with a nanosecond laser (3 ns pulse, 532 nm wavelength), using a Galvano scanner to allow rapid scanning of the laser beam across samples (5–6 s well^−1^). Laser irradiation was arranged such that each location in the well received a single‐laser pulse, apart from those areas where adjacent illumination spots overlapped to ensure full coverage of the sample.

##### Assessment of Cell Viability

Determination of the cytotoxic effect of the various AuNP formulations and the impact of laser treatment was conducted by measuring the relative cell viability using the CellTiter Glo luminescent cell viability assay (Promega, Madison, WI, USA) after allowing the cells to rest in an incubator (37 °C, 5% CO_2_) for 4 h post‐treatment. This assay allowed a measurement of the number of viable cells by quantifying the adenosine triphosphate (ATP) present after cell lysis, which was directly correlated to the metabolic activity of cells and thus the number of live cells present. As required by the manufacturer, the old culture medium was first removed and replaced by fresh culture medium. Next, this was complemented by an equal volume of CellTiter Glo reagent and the plate was shaken on an orbital shaker (120 rpm) for 10 min at room temperature. Upon transferring the cell lysates to an opaque white 96‐well plate (Greiner Bio‐One, Kremsmünster, Austria), the luminescent signal was recorded using a GloMax microplate reader (Promega, Madison, WI, USA). In the last step, the relative cell viability was calculated by dividing the luminescent signal intensity of the samples by the average luminescence of a technical triplicate of nontreated controls. For cells treated with FOL‐containing AuNPs, the viabilities were measured relative to cells incubated with the lowest relevant concentration of (DOX‐)FOL‐AuNPs (no laser treatment), since the presence of FOL was consistently observed to increase ATP levels, likely due to its role in ATP metabolism^[^ ^100^, ^101^, ^102^ ^]^ and in spite of covalent binding to the nanoparticle surface. This increase was observed to be constant, even when further increasing the concentration of (DOX‐)FOL‐AuNPs.

##### Assessment of Apoptosis

A proxy for the apoptotic levels induced by the various AuNP formulations combined with the laser treatment was obtained by measuring the caspase 3/7 activity using the Caspase‐Glo luminescent assay (Promega, Madison, WI, USA) after allowing the cells to rest in an incubator (37 °C, 5% CO_2_) for 4 h post‐treatment. This assay allowed a measurement of the induced apoptotic cell death pathways in cells by quantifying the caspase 3/7 present after cell lysis, where the signal intensity was directly correlated to the apoptotic activity of cells. As required by the manufacturer, the old culture medium was first removed and replaced by fresh culture medium. Next, this was complemented by an equal volume of Caspase‐Glo reagent and the plate was shaken on an orbital shaker (120 rpm) for 1 min at room temperature before being incubated for 2 h at room temperature (shielded from light). Upon transferring the cell lysates to an opaque white 96‐well plate (Greiner Bio‐One, Kremsmünster, Austria), the luminescent signal was then recorded using a GloMax microplate reader (Promega, Madison, WI, USA). In the last step, the relative caspase 3/7 activity was calculated by dividing the luminescent signal intensity of the samples by the average luminescence of a technical triplicate of nontreated controls. Normalization of the relative caspase 3/7 activities was obtained by dividing the relative activities by the mean relative cell viabilities as obtained using the CellTiter‐Glo assay (Section 5) to account for differences in cell densities 4 h post‐treatment due to nonapoptotic induced cell deaths.

##### Flow Cytometric Determination of AuNP Attachment/Internalization

Since the AuNP formulations also included functionalization with the borondipyrromethene‐based fluorescent dye BDP 630/650 (Lumiprobe, Hannover, Germany), the attachment and/or uptake of those nanoparticles by A549/SKOV3 cells was studied using flow cytometry. Following the same protocol described in Section 5, the viable cells were first stained with 50 μl well^−1^ of 0.5 × 10^−6^ M calcein AM (Gibco, Grand Island, NY, USA) in DPBS(−/−) for 15 min at room temperature. This was followed by single‐washing step using 100 μl of DPBS(−/−) and detachment of the cells from their plates by addition of 50 μl well^−1^ of trypsin–EDTA (0.25%) (Gibco, Grand Island, NY, USA) and incubation for 3–5 min (37 °C, 5% CO_2_). The trypsin was then neutralized by adding 50 μl of fresh culture medium, allowing the cells to be transferred to a U‐bottomed 96‐well plate. Next, the cells were spun down in a plate centrifuge for 5 min at 300 RCF, after which the supernatant was removed and the cells were resuspended in 100 μl flow buffer [DPBS(−/−), 1% BSA, 0.1% sodium azide]. Once resuspended, the samples were measured using a CytoFLEX flow cytometer (Beckman Coulter, Krefeld, Germany), where calcein AM and BDP 630/650 were excited by 488 nm and 638 nm lasers, and emission was detected using 525/40 nm and 660/20 nm bandpass filters, respectively. The gating strategy entailed gating 10.000 living cells for the evaluation of AuNP attachment/uptake in the allophycocyanin (APC) channel by excluding calcein AM negative cells––based on unstained control––in the singlet population. FlowJo software (Tree Star Inc., Ashland, OR, USA) was used to perform the data analysis.

##### Study of Cellular AuNP Localization by TEM

A more in‐depth investigation of the localization of the various key AuNP formulations incubated with A549/SKOV3 cells at a concentration corresponding to clear differences between the formulations (3 nM) was achieved by taking TEM images. Here, cells were grown and treated as previously described in Section 5, which was then followed by a washing step using DPBS(−/−) and cell detachment using Trypsin‐EDTA (cf. Section 5). Upon transferring the resuspended cells to 5 mL Eppendorf tubes (Hamburg, Germany), the cells were centrifuged for 5 min at 300 RCF. Subsequently, the supernatant was removed, and the cells were fixed using 2.5% glutaraldehyde (electron microscopy grade) in 0.1 M sodium cacodylate buffer at pH 7.2, fixing them overnight at 4 °C. Cells were postfixed for 2 h in 1% osmium tetroxide (OsO_4_) in a 0.033 M veronal acetate buffer. After performing a washing step with 0.05 M veronal acetate buffer (pH 7.4), cells were stained with 1% tannic acid (1 h at RT) and the samples were washed thoroughly with veronal acetate, followed by dehydrating using an ethanol gradient starting from 50% to 100% ethanol. Dehydration was continued with propylene oxide for 30 min. Eventually, the cells were embedded in EMBed 812 resin (EMBed‐812 Kit, E14120). Using an ultramicrotome (EM UC7, Leica, Wetzlar, Germany), ultrathin sections having a gold interference color were obtained. These ultrathin sections were stained with lead citrate and examined in a Tecnai G2 Spirit Bio TWIN microscope (Thermo Fisher Scientific, FEI Europe BV, Eindhoven, The Netherlands) at 120 kV.

##### VNB Visualization

The propensity of AuNP formulations incubated with A549/SKOV3 cells to generate VNBs was investigated by irradiating cells seeded at a density of 3 × 10^6^ cells/well in a regular six‐well plate 24 h prior to the experiment. The cells were incubated with 1.5 mL of AuNPs (cf. Section 5) with ≈7 ns pulsed laser light tuned to 532 nm with a beam diameter of 131 μm (Opolette HE 355 LD, OPOTEK Inc., Carlsbad, CA, USA). A concentration of 4 nM was chosen for the various AuNPs since this corresponded to the most effective treatments. An electronic pulse generator (BNC575, Berkeley Nucleonics Corporation, San Rafael, CA, USA) was used to generate the laser pulses. Laser energies were registered using a J‐25MB‐HE&LE energy meter (Energy Max‐USB/RS sensors, Coherent Inc., Santa Clara, CA, USA). Visualization of VNBs was achieved using dark‐field microscopy, where the enhanced scatter of the VNBs generated bright white spots.^[^ ^46^ ^]^ This entire setup was developed in house. Images synchronized to the laser pulse—before and after—were captured via a Prime BSI sCMOS camera (Teledyne Photometrics, Tucson, AZ, USA). This allowed for the counting of individual VNBs in ImageJ.^[^ ^103^, ^104^ ^]^ Low magnification (10×), reduced dark‐field illumination intensity, and adjustments in global image brightness and contrast were selected to improve the contrast between VNBs and the background signal from cells and AuNPs.

##### Fluorimetric Determination of Doxorubicin Release

To assess the ability to release DOX from the surface of DOX‐AuNPs and DOX‐FOL‐AuNPs upon laser irradiation, 10 μl drops of 50 nM stock suspensions (= 50 μM DOX upon 100% release) were pipetted into the center of wells in a 96‐well plate and irradiated with pulsed laser light. Nonirradiated mPEG‐AuNPs were used as a blank. Afterward, 140 μl of HyClone Pure water was added to the wells and the contents were transferred to 1.5 mL Eppendorf tubes, which were then centrifuged for 30 min at 13.500 RCF and 4 °C. Next, 60 μl of the supernatant was transferred to an opaque black 96‐well plate suitable for fluorimetry. In this well plate, a series of DOX standards (0, 0.05, 0.1, 0.25, and 0.5 μM) in HyClone Pure water, as well as noncentrifuged AuNP formulations corresponding to all included treatments at equal diluted states, were added. Using a Victor 31 420 Multilabel Counter (PerkinElmer, Waltham, MA, USA), DOX was excited at 485 nm and the fluorescent intensity (A.U.) was measured at 590 nm.^[^ ^105^ ^]^ The noncentrifuged samples were used to approximate the amount of DOX that got broken down due to the laser treatments themselves, as induced by photothermal effects of the AuNPs. After correcting for the blank, linear regression was applied to obtain a standard curve, which was then used to calculate the concentration of released DOX in every condition, which was corrected for the blank and broken‐down DOX for the various laser treatments. Both the dilution factor and differences in stock nanoparticle concentrations were taken into account. All nanoparticle samples were present as technical triplicates for three different nanoparticle batches per formulation. Standard concentrations of free DOX were present as a technical triplicate.

##### Induced Toxicity, Its Spatial Control, and Endosomal Disruption Visualized by Confocal Microscopy

Following the incubation of 96‐well plates containing A549 and SKOV‐3 cells incubated with DOX‐AuNPs, DOX‐FOL‐AuNPs, or no nanoparticles (see Section 5), sample wells were irradiated either completely (Section 2.4) or according to a pattern depicting the logo of Ghent University (Section 2.6), both with a pulsed laser at a fluence of 1.1 J cm^−2^. After the laser treatment, the cells were left in an incubator (37 °C, 5% CO_2_) for 4 h to let the cytotoxicity take effect. Subsequently, the cells were stained with calcein AM (Invitrogen, Waltham, MA, USA) to visualize live cells and stain the cytoplasmic space, and TO‐PRO3 iodide (Invitrogen, Waltham, MA, USA), to stain the nuclei of apoptotic/dead cells, for 20 min at room temperature. Removal of the staining solution was followed by the addition of 200 μl of fresh culture medium. Visualization of the samples was obtained using a Nikon A1R HD confocal laser scanning microscope (Nikon, Tokyo, Japan) with a Plan Apo 10× air lens (NA 0.45, WD 4000 μm). The 488 nm and 637 nm laser lines were used for calcein AM and TO‐PRO3 iodide, respectively. Fluorescence was detected through a 525/50 nm (MHE57030) and 700/75 nm (MHE57070) emission filters on an A1‐DUG‐2 GaAsP multidetector unit (Nikon, Tokyo, Japan). A scanning speed of 0.25 FPS was used to unidirectionally scan and acquire the channels with a Galvano scanner in a sequential manner. Representative single images (1024 × 1024 pixels) of the induced toxicity were taken. Pixel size was set to 1.44 μm pixel^−1^, and pinhole size was set at 20.43 μm (= 1.1 AU). For the spatial‐induced toxicity experiment, large field‐of‐view Z‐stacked images (3.05 μm steps) of 7 × 7 squares (1024 × 1024 pixels each) were acquired with 30% overlap and subsequently stitched together using a blending algorithm. The pixel size was set to 1.22 μm pixel^−1^, with the pinhole being set to 20.43 μm (= 0.6 AU). Overview images of the spatial induced toxicity for other samples were taken using identical settings, apart from the pixel size and image resolution that were changed to 3.95 μm pixel^−1^ and 256 × 256 pixels, respectively. Pinhole size was set at 20.43 μm (= 1.1 AU).

Deviating slightly from the protocol described in Section 5, SKOV‐3 and A549 cells were incubated with 2 nM of DOX‐(FOL‐)AuNPs and 4 nM of (FOL‐)AuNPs in the presence of 400 μg ml^−1^ of calcein in order to perform a calcein release assay.^[^ ^63^ ^]^ Then, a laser pulse fluence of 0.13 J cm^−2^ was selected for all laser‐treated wells. Immediately following the laser irradiation, the cells were stained with Hoechst 33 342 (Invitrogen, Waltham, MA, USA) to stain the nuclei for 20 min at room temperature. Again, 200 μl of fresh culture medium replaced the staining solution upon removal. Using the aforementioned confocal microscope, now a Plan Apo VC 20× air objective (NA 0.8, WD 1000 μm) was used. Here, the 405 nm and 488 nm laser lines were used to excite Hoechst 3342 and calcein, respectively. Fluorescence was detected through 450/50 nm (MHE57010) and 525/50 nm (MHE57030) emission filters on an A1‐DUG‐2 GaAsP multidetector unit (Nikon, Tokyo, Japan). Once more, a scan speed of 0.25 FPS was used to unidirectionally scan and acquire the channels with a galvano scanner in a sequential manner. Single images of 1024 × 1024 pixels were taken. Pixel size was set to 0.29 μm pixel^−1^, and pinhole size was set at 39.59 μm (= 2.7 AU).

In ImageJ,^[^ ^103^, ^104^ ^]^ the images were processed. For the spatially selective treatment, a Z‐projection of the average pixel intensity was performed. Endosomal escape events were manually quantified, counting at least 500 cells for each treatment spread over three biological replicates each consisting of three technical replicates.

##### Statistical Analysis

All data were represented by their mean ± standard deviation, unless stated otherwise. GraphPad Prism v.8.0.0 (GraphPad Software, Boston, MA, USA) was used for the graphical representations and statistical tests, which are further specified in the figure captions.

## Conflict of Interest

K.B. and S.C.D.S. declare financial interest in the company Trince.

## Author Contributions

**Ilia Goemaere**: conceptualization (equal); formal analysis (lead); investigation (lead); methodology (lead); visualization (lead); writing—original draft (lead); writing—review and editing (lead). **Anna Cielo**: investigation (supporting); writing—review and editing (supporting). **Raffaella Daniele**: investigation (supporting); writing—review and editing (supporting). **Francesca Mastrotto**: conceptualization (equal); investigation (supporting); writing—review and editing (supporting). **Stefaan C. De Smedt**: supervision (supporting); writing—review and editing (supporting). **Winnok H. De Vos**: conceptualization (equal); funding acquisition (supporting); supervision (supporting); writing—review and editing (supporting). **Stefano Salmaso**: conceptualization (equal); writing—review and editing (supporting). **Kevin Braeckmans**: conceptualization (equal); funding acquisition (lead); Supervision (lead); writing—review and editing (lead).

## Supporting information

Supplementary Material

## Data Availability

The data that support the findings of this study are available from the corresponding author upon reasonable request.
