# Supplementary material for: Nanosecond Laser Pulses Facilitating Efficient and Specific Cell Killing with Doxorubicin‐Loaded Gold Nanoparticles Targeted to the Folate Receptor
Source: Small Sci. 2024 Dec 19;5(2):2400234. doi: 10.1002/smsc.202400234 (PMC11934907; doi:10.1002/smsc.202400234)
Supplement: Supplementary file 1 — Supplementary Material [file SMSC-5-2400234-s001.pdf]

Supporting information

Nanosecond laser pulses facilitate efficient and specific cell killing with doxorubicin-loaded gold nanoparticles targeted to the folate receptor

Ilia Goemaere [a,b], Anna Cielo [c], Raffaella Daniele [c], Francesca Mastrotto [c], Stefaan C. De Smedt [a], Winnok H. De Vos [b, #], Stefano Salmaso [c,#] and Kevin Braeckmans [a,#,*]

[a] Laboratory of General Biochemistry and Physical Pharmacy, Faculty of Pharmaceutical Sciences, Ghent University, Ottergemsesteenweg 460, 9000 Ghent, Belgium

[b] Laboratory of Cell Biology and Histology, Department of Veterinary Sciences, University of Antwerp, Universiteitsplein 1, 2610 Wilrijk, Belgium

[c] Department of Pharmaceutical and Pharmacological Sciences, University of Padova, Via Francesco Marzolo 5, 35131 Padova, Italy

[#]Shared senior authorship

*Correspondence: Kevin.Braeckmans@ugent.be; Tel.: +32-9-2648098; Fax: +32-9-2648189

Table S1. Summary of the Z-average hydrodynamic diameter (size) and polydispersity index (PDI) of the AuNPs, DOX-coated AuNPs and DOX-FOL-coated AuNPs in HyClone Pure water, and after 4h incubation in SKOV-3 FCM and A549 FCM. The zeta potential (Z.P.) was additionally determined in HyClone Pure water. Represented by the mean ± standard deviation of three replicates.

	Medium	Size [nm]	PDI	Z.P. [mV]
**AuNP**	HyClone	25.0 ± 0.1	0.186 ± 0.016	-25.5 ± 3.1
	FCM SKOV-3	44.5 ± 0.4	0.201 ± 0.009	N.A.
	FCM A549	51.1 ± 0.6	0.259 ± 0.034	N.A.
**DOX-AuNP**	HyClone	25.8 ± 0.7	0.485 ± 0.023	-27.0 ± 5.0
	FCM SKOV-3	20.1 ± 0.3	0.427 ± 0.013	N.A.
	FCM A549	20.0 ± 0.3	0.438 ± 0.009	N.A.
**DOX-FOL-AuNP**	HyClone	21.1 ± 0.3	0.442 ± 0.006	-20.9 ± 0.5
	FCM SKOV-3	18.9 ± 0.6	0.378 ± 0.005	N.A.
	FCM A549	19.6 ± 1.3	0.378 ± 0.048	N.A.

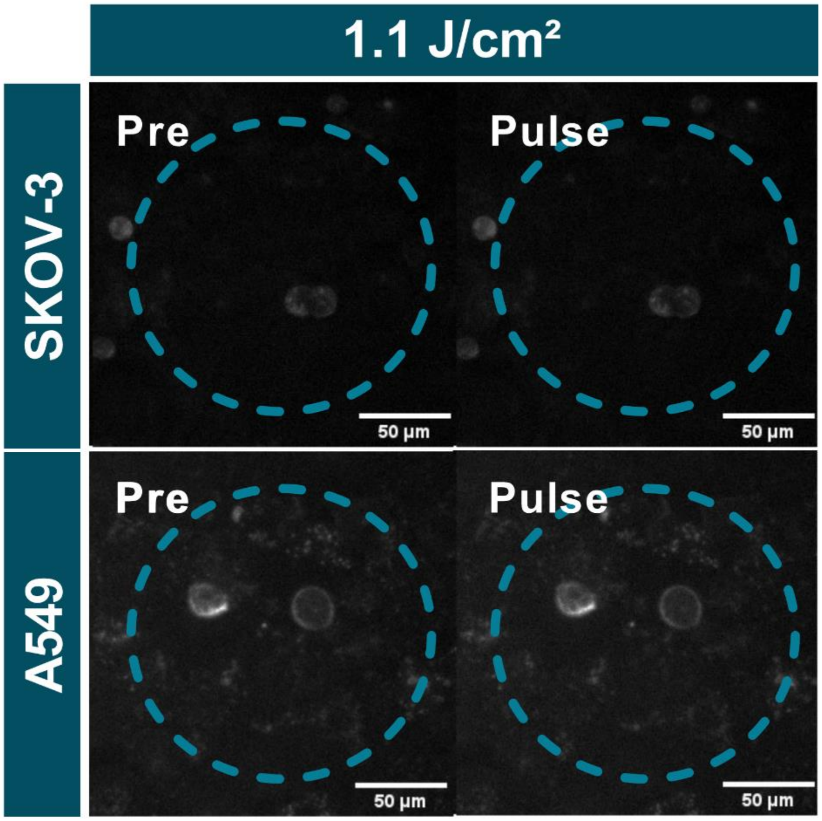

Figure S1. Visualization of VNB-generation per cell type. Dark-field microscopy images to visualize VNB-formation at the highest laser pulse fluence used in this study (1.1 J/cm²), where the irradiated zone is delineated with a dashed blue circle. No VNBs were observed for SKOV-3 and A549 cells that were not incubated with nanoparticles. Scale bars = 50 μm.

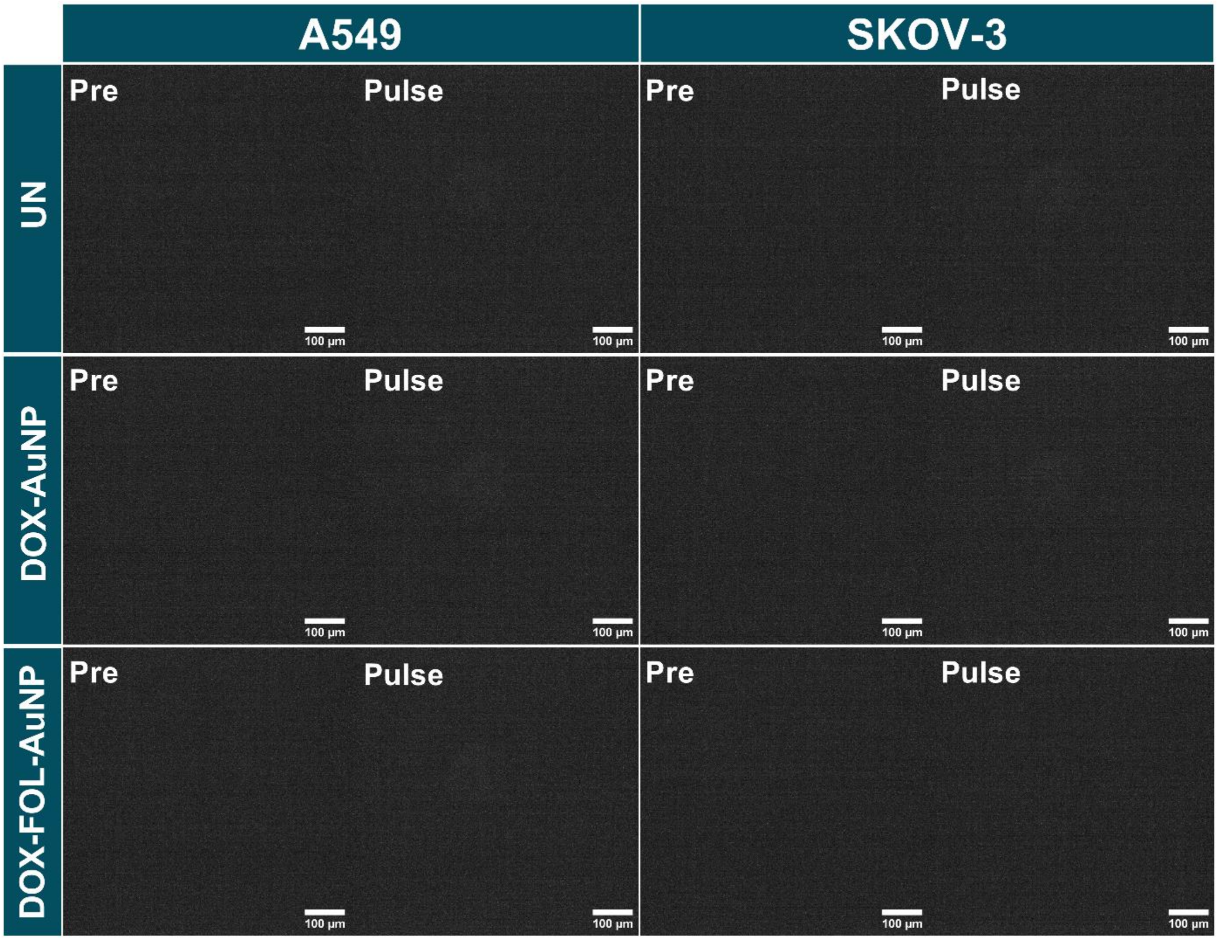

Figure S2. Visualization of bright light bursts per cell type and AuNP formulation without dark-field illumination. Samples were irradiated at the highest laser pulse fluence used in this study (1.1 J/cm²). In the absence of dark-field illumination, no bright bursts of light were observed for SKOV-3 and A549 cells, whether or not they were incubated with nanoparticles, confirming that DOX fluorescence did not cause the bright bursts of light associated with VNB formation. Scale bars = 100 µm.

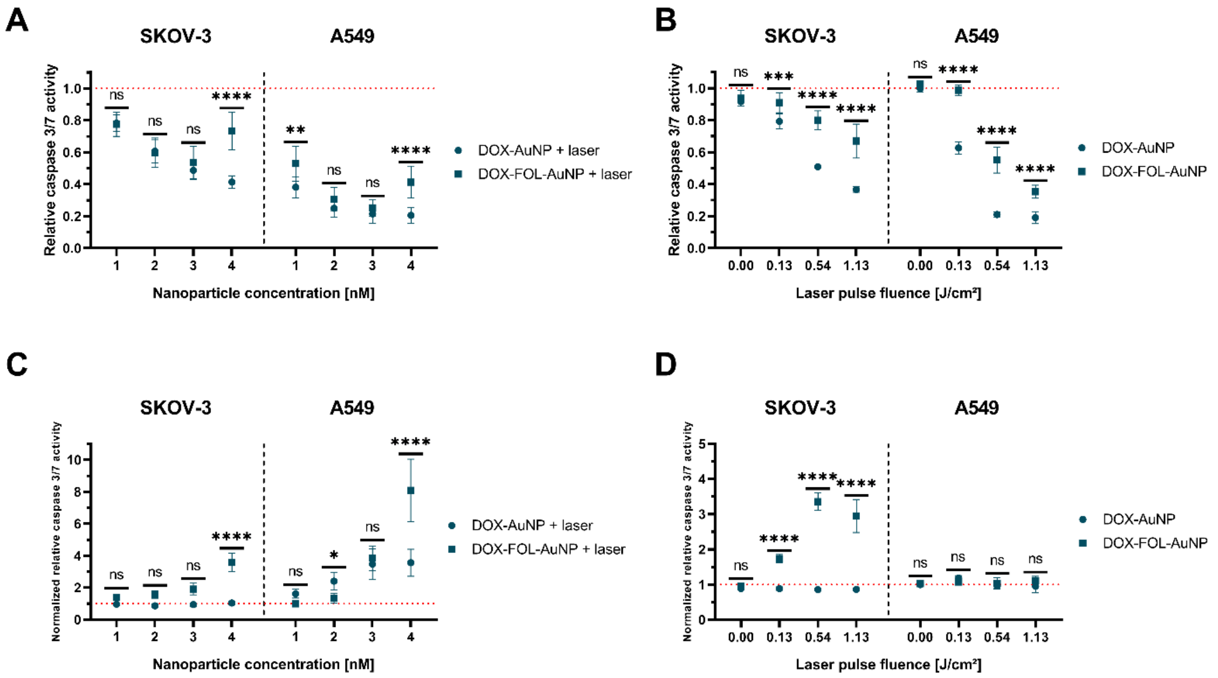

Figure S3. Apoptosis induced by DOX-AuNPs and DOX-FOL-AuNPs upon pulsed laser irradiation. A) Effect of various concentrations of DOX-AuNPs (circles) and DOX-FOL-AuNPs (squares) on caspase 3/7 activity relative to an untreated control – as measured by a Caspase-Glo® 3/7 assay 4 hours post-treatment – upon 1.1 J/cm^2 pulsed laser irradiation of SKOV-3 and A549 cells. B) Relative caspase 3/7 activities for different laser pulse fluences and fixed nanoparticle concentrations (4 nM on SKOV-3 and 2 nM on A549 cells). C) Normalization of caspase 3/7 activity presented in (A) by dividing the data by the mean relative viabilities as obtained by a CellTiter-Glo® assay (Figure 4A main manuscript). D) Normalization of caspase 3/7 activity presented in (C) by dividing the data by the mean relative viabilities as obtained by a CellTiter-Glo® assay (Figure 4C main manuscript). Dotted red line represents the untreated baseline. Two-way ANOVAs with Tukey-corrected multiple comparison tests were performed to determine statistically different results between the two formulations. Data are represented as the mean ± standard deviation of three biological replicates, each consisting of three technical replicates. * $p < 0.05$, ** $p < 0.01$, *** $p < 0.001$, **** $p < 0.0001$.

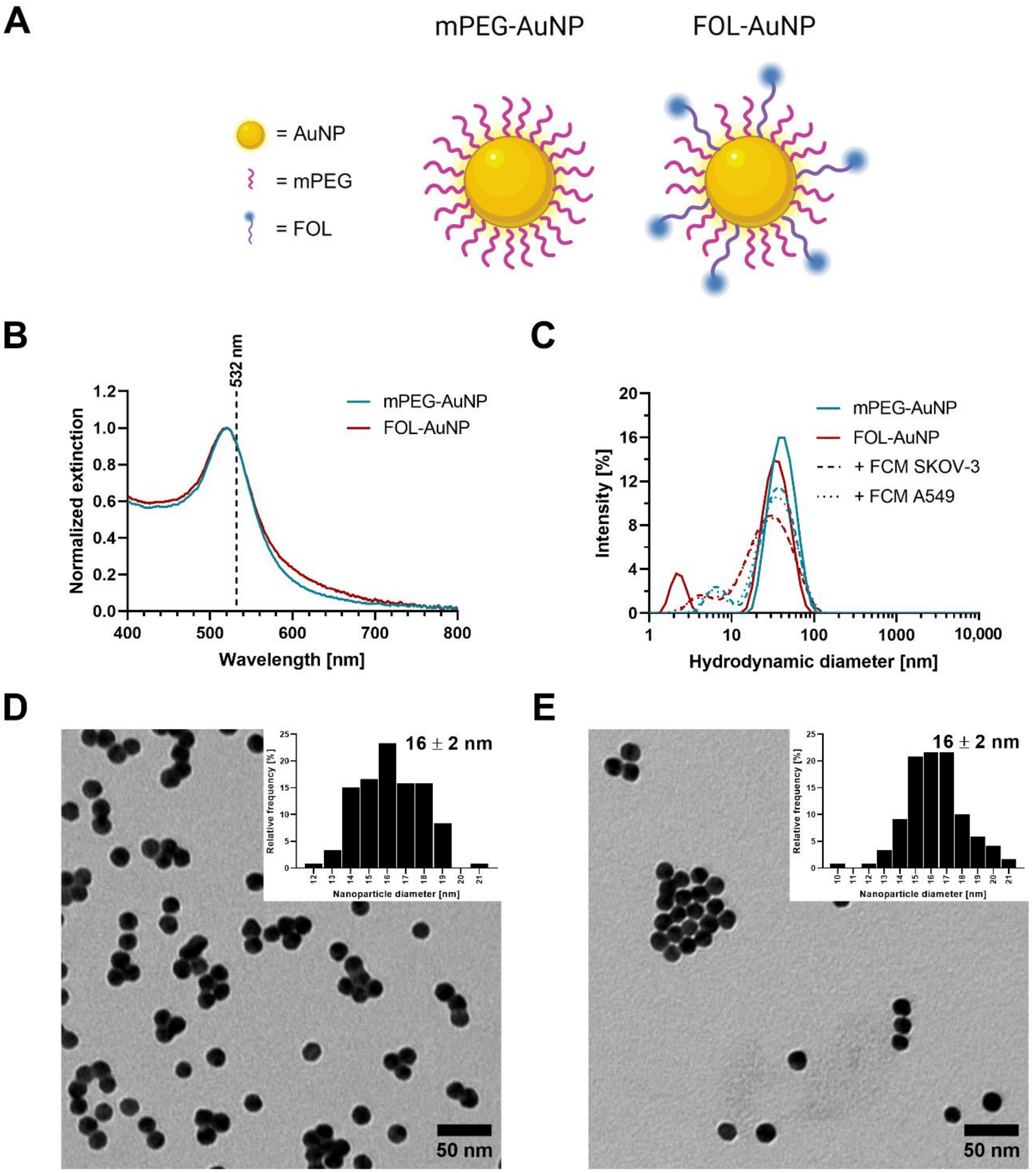

Figure S4. Characterization of (FOL-coated) gold nanoparticles (AuNPs). A) Schematic overview of the mPEG-AuNPs and FOL-AuNPs used to study the photothermally induced toxicity. B) Normalized UV/Vis spectra of mPEG-AuNPs (blue) and FOL-AuNPs (red) C) Intensity size distributions of the AuNP formulations in HyClone Pure water (full line), and after 4h incubation in FCM for SKOV-3 cells (dashed) or A549 cells (dotted). For both mPEG-AuNPs (blue) and FOL-AuNPs (red). D&E) Representative TEM images of mPEG-AuNPs (D) and FOL-AuNPs (E). Scale bars = 50 nm. Size distribution and mean diameter ± standard deviation is displayed in the topright corner.

Table S2. Summary of the Z-average hydrodynamic diameter (size) and polydispersity index (PDI) of the mPEG-AuNPs and FOL-coated AuNPs in HyClone Pure water, and after 4h incubation in SKOV-3 FCM and A549 FCM. The zeta potential (Z.P.) was additionally determined in HyClone Pure water. Represented by the mean ± standard deviation of three replicates.

	**Medium**	**Size [nm]**	**PDI**	**Z.P. [mV]**
**mPEG-AuNP**	HyClone	25.8 ± 0.7	0.284 ± 0.034	-21.0 ± 0.1
	FCM SKOV-3	25.6 ± 0.3	0.299 ± 0.008	N.A.
	FCM A549	25.1 ± 0.1	0.301 ± 0.001	N.A.
**FOL-AuNP**	HyClone	20.9 ± 0.2	0.416 ± 0.003	-21.8 ± 2.2
	FCM SKOV-3	19.7 ± 0.4	0.403 ± 0.003	N.A.
	FCM A549	19.2 ± 0.2	0.371 ± 0.040	N.A.

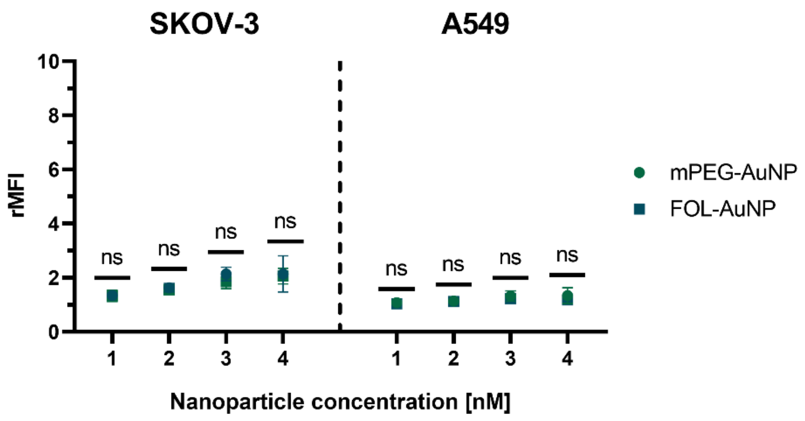

Figure S5. Nanoparticle-cell interactions evaluated for AuNPs and FOL-AuNPs. Fluorescently labeled mPEG-AuNPs (green circles) and FOL-AuNPs (blue squares) were incubated with SKOV-3 cells and A549 cells. Their amount per cell was quantified by flow cytometry as the median fluorescent intensity relative to untreated cells (rMFI). Data are represented by the mean ± standard deviation of three biological replicates, each consisting of three technical replicates. Two-way ANOVAs with Tukey-corrected multiple comparison tests were performed to determine statistically different results between mPEG-AuNP and FOL-AuNP rMFIs . * $p < 0.05$, ** $p < 0.01$, *** $p < 0.001$, **** $p < 0.0001$.

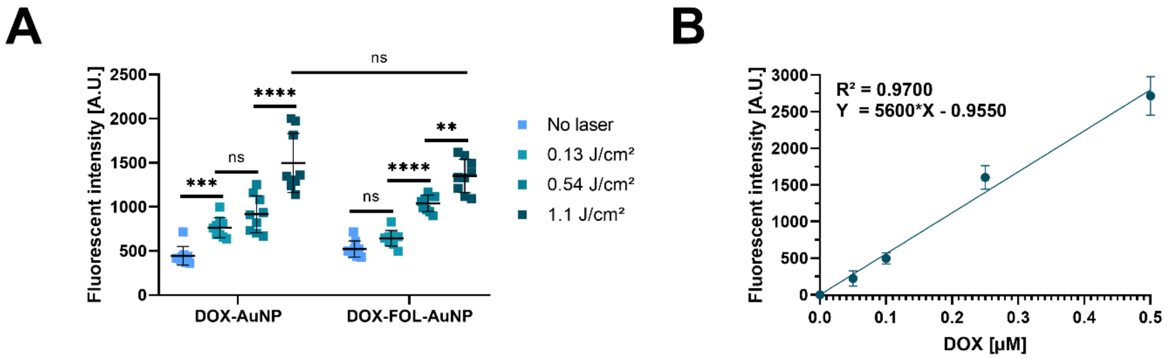

Figure S6. Analysis of DOX-release induced by laser irradiation using fluorimetry. A) Blank- and broken-down-DOX-corrected fluorescent intensities (A.U.) of DOX released by DOX-AuNPs and DOX-FOL-AuNPs treated without or with various laser pulse fluences relevant to this study. B) Calibration curve – corrected for the blank and fitted using linear regression – corresponding to various concentrations of free DOX diluted in HyClone Pure water, identical to the nanoparticle formulations, spanning the range of possible DOX-concentrations upon release from the nanoparticles. Two-way ANOVAs with Tukey-corrected multiple comparison tests were performed to determine statistically different results within groups and Sidak-corrected multiple comparisons tests to test statistically different results between groups. Data are represented by the mean ± standard deviation of A) three functionalized AuNP batches, each consisting of three technical replicates, and B) three technical replicates. * $p < 0.05$, ** $p < 0.01$, *** $p < 0.001$, **** $p < 0.0001$.

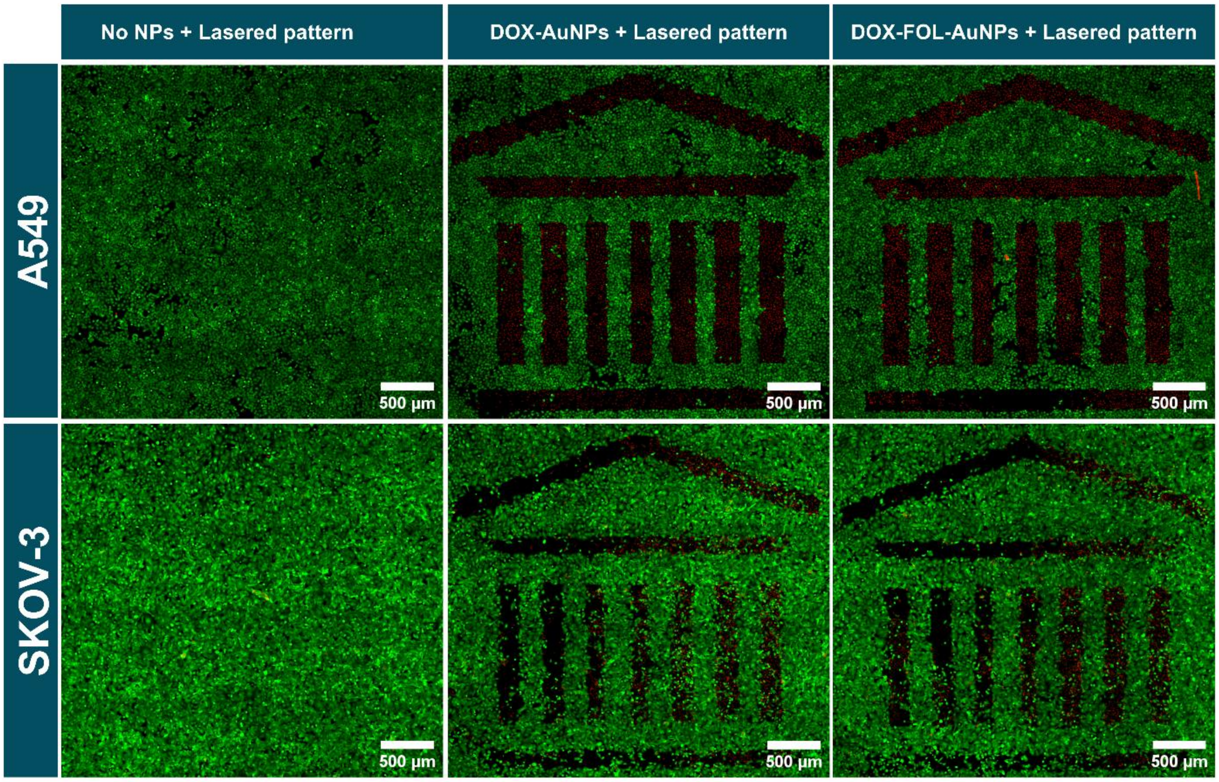

Figure S7. Spatially selective induced cytotoxicity by DOX-AuNPs and DOX-FOL-AuNPs upon pulsed laser irradiation. A549 and SKOV-3 cells without and with DOX-AUNPs irradiated at 1.1 J/cm^2 according to a pattern resembling the Ghent University logo. Viable cells were stained with Calcein AM (green), while nuclei of dead cells were stained with TO-PRO3 iodide (red). Scale bars = 500 µm.
